# Supplementary material for: Breaking solvation dominance of ethylene carbonate via molecular charge engineering enables lower temperature battery
Source: Nat Commun. 2023 Dec 14;14:8326. doi: 10.1038/s41467-023-43163-9 (PMC10721867; doi:10.1038/s41467-023-43163-9)
Supplement: Supplementary file 1 — Supplementary Information [file 41467_2023_43163_MOESM1_ESM.pdf]

Supporting Information

Breaking Solvation Dominance of Ethylene Carbonate via Molecular Charge Engineering Enables Lower Temperature Battery

Yuqing Chen[1], Qiu He[2], Yun Zhao[3], Wang Zhou[1], Peitao Xiao[4], Peng Gao[1], Naser Tavajohi[5], Jian Tu[6], Baohua Li[3], Xiangming He[7], Lidan Xing[8], Xiulin Fan[9], Jilei Liu[1*]

[1]*College of Materials Science and Engineering, Hunan Joint International Laboratory of Advanced Materials and Technology of Clean Energy, Hunan Province Key Laboratory for Advanced Carbon Materials and Applied Technology, Hunan University, Changsha 410082, People's Republic of China*

[2]*College of Materials Science and Engineering, Sichuan University, Chengdu 610065, P. R. China.*

[3]*Institute of Materials Research, Tsinghua Shenzhen International Graduate School, Tsinghua University, Shenzhen518055, China*

[4] *College of Aerospace Science and Engineering, National University of Defense Technology, Changsha 410073, China*

[5] *Department of Chemistry, Umeå University, Umeå, 90187, Sweden*

[6]*LI-FUN Technology Corporation Limited, Zhuzhou, 412000, Hunan, China*

[7]*Institute of Nuclear and New Energy Technology, Tsinghua University, Beijing 100084, China*

[8]*Engineering Research Center of MTEES (Ministry of Education), Research Center of BMET (Guangdong Province), Engineering Lab. of OFMHEB (Guangdong Province), Key Lab. of ETESPG (GHEI), And Innovative Platform for ITBMD (Guangzhou Municipality), School of Chemistry, South China Normal University, Guangzhou, 510006, China*

[9]*State Key Laboratory of Silicon Materials, School of Materials Science and Engineering, Zhejiang University, Hangzhou 310027, China*

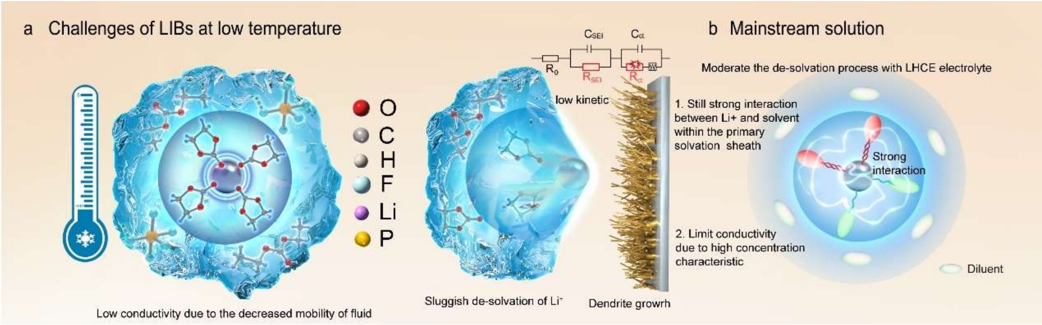

Supplementary Fig.1| a, Challenges of lithium-ion batteries operating at low temperature. b, The main strategy of LHCE electrolyte towards low temperature performance.

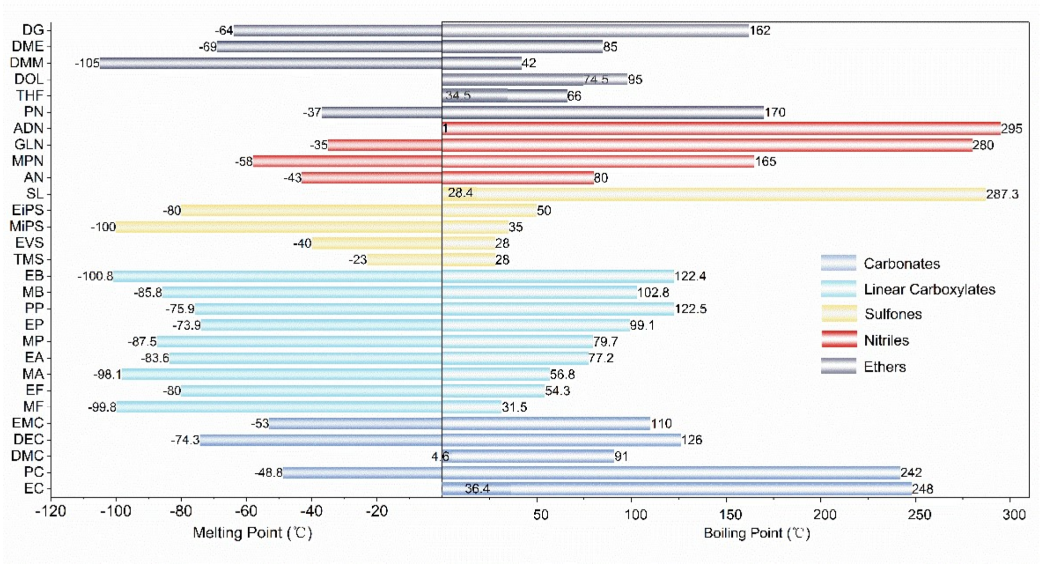

Supplementary Fig.2| The melting point and boiling point of various solvents, including carbonates, linear carboxylates, sulfones, nitriles, and ethers.

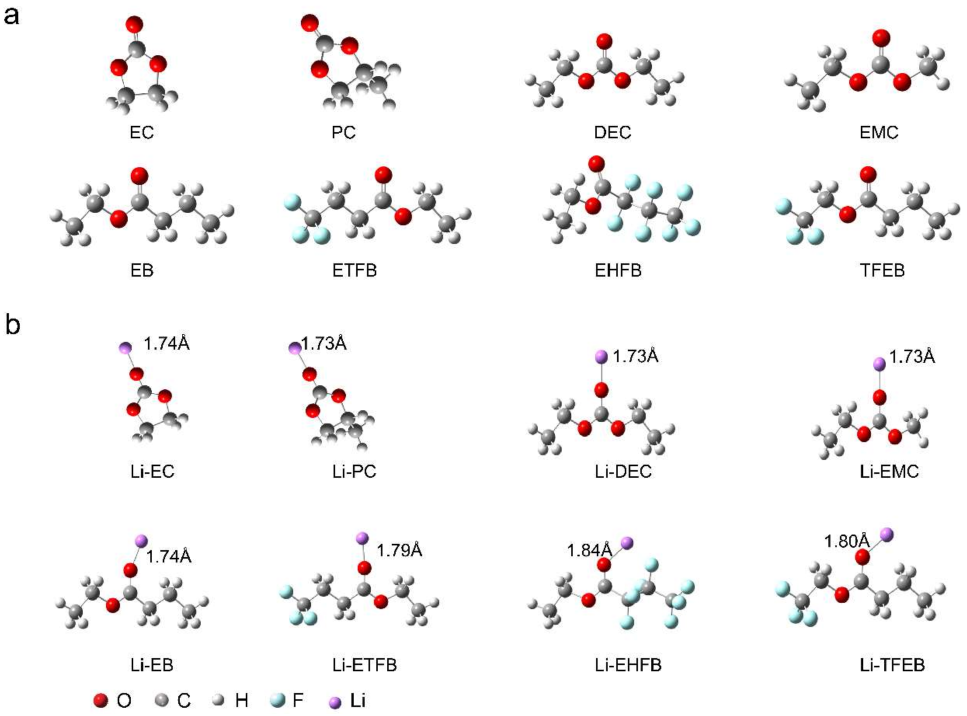

Supplementary Fig.3| a, The molecular structures of the liquid solvents used in this work. b, Optimized structures of Li coordinates with different solvents. The red, dark grey, light grey, cyan, and purple ball represents O atom, C atom, H atom, F atom, and Li atom, respectively.

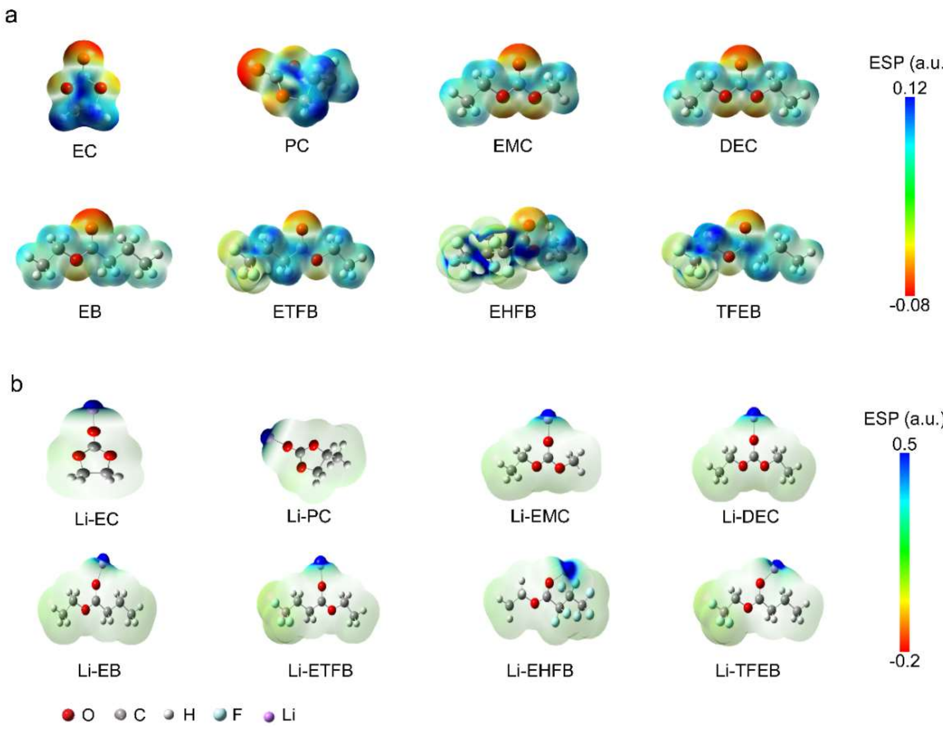

Supplementary Fig.4| Electrostatic potential (ESP) of EC, PC, EMC, DEC, EB, ETFB, EHFB, and TFEB molecules (a) and corresponding Li^+ coordinated structure (b). The red, dark grey, light grey,

cyan, and purple ball represents O atom, C atom, H atom, F atom, and Li atom, respectively.

Abbreviation List

Abbreviation	Full Name
EC	Ethylene carbonate
PC	Propylene carbonate
DMC	Dimethyl Carbonate
DEC	Diethyl carbonate
EMC	Ethyl Methyl Carbonate
EF	Ethyl Formate
EA	Ethyl Acetate
MP	Methyl Propanoate
EP	Ethyl Propionate
PP	Propyl Propanoate
MB	Methyl Butyrate
EB	Ethyl Butyrate
ETFB	4,4,4-Ethyl trifluorobutyrate
EHFB	Ethyl heptafluorobutyrate
TFEB	2,2,2-trifluoroethyl butyrate
THF	Tetrahydrofuran
DME	1,2-dimethoxyethane
DG	Dimethyl Glycol

Supplementary Table 1: Carbonyl oxygen charge of various solvents.

Solvent	EC	PC	DEC	EMC	EB	ETFB	EHFB	TFEB
Charge of oxygen	−0.33	−0.331	−0.302	−0.308	−0.288	−0.281	−0.207	− 0.27

Supplementary Table 2: Frontier molecular orbital energy and electron energy of solvent and coordinated solvent, and the corresponding binding energy and Li band length.

Molecular	Uncoordinated			Coordinated				Binding Energy (eV)
	Electron Energy (eV)	HOMO (eV)	LUMO (eV)	Electron Energy(eV)	HOMO (eV)	LUMO (eV)	Li band length(Å)	
EC	−9319	−8.02	−0.28	−9520	−12.65	−5.02	1.74	−2.24
PC	−10390	−8.36	−0.31	−10590	−12.83	−4.95	1.73	−2.31
DEC	−11493	−8.05	0.07	−11693	−12.47	−5.23	1.73	−2.09
EMC	−10423	−8.13	0.07	−10623	−12.67	−5.30	1.73	−2.02
EB	−10515	−8.14	−0.33	−10716	−13.05	−5.11	1.74	−2.18
ETFB	−18619	−8.31	−0.38	−18819	−13.13	−5.37	1.79	−2.86
EHFB	−29422	−8.82	−1.54	−29622	−13.48	−6.11	1.84	−1.72
TFEB	−18618	−8.47	−0.53	−18818	−13.47	−5.32	1.80	−1.83

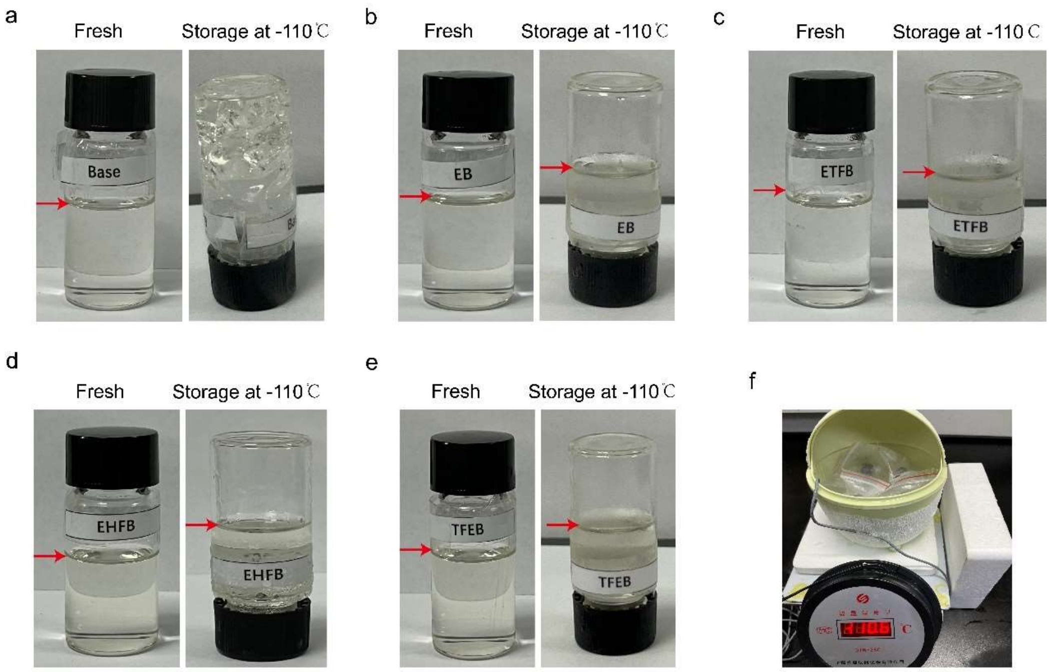

Supplementary Fig.5| a-e Digital images of different electrolytes before and after keeping at −50 °C for 30 min. f, Cold trap device for electrolyte low temperature storage test as well as the conductivity.

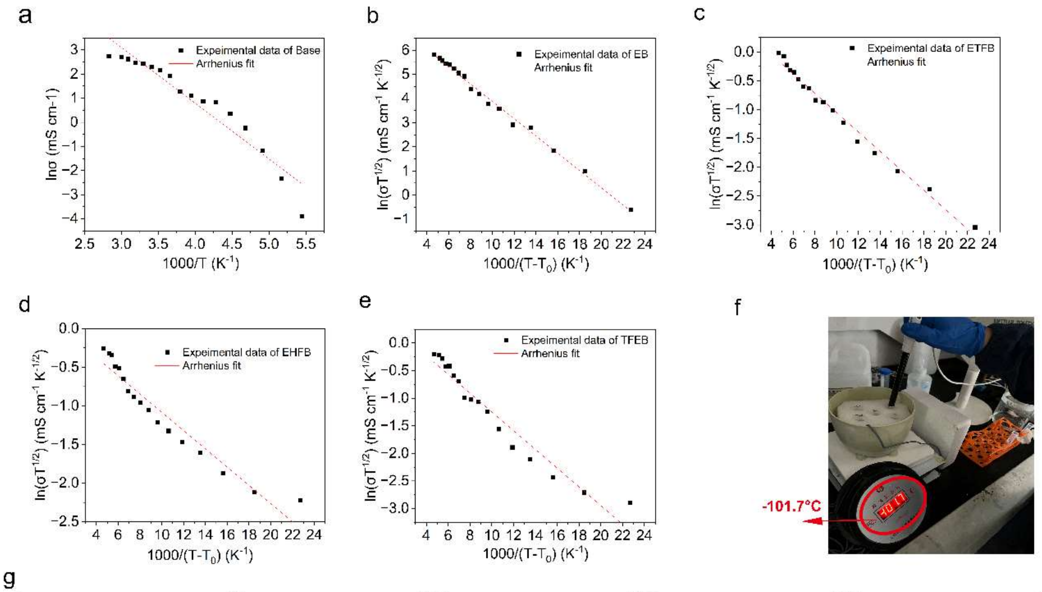

g

Base

	A	B
1	Equation	y = a + b*x
2	Plot	lnσ
3	Weight	No Weighting
4	Intercept	10.02342 ± 0.68303
5	Slope	-2.30825 ± 0.17054
6	Residual Sum of Squares	4.40383
7	Pearson's r	-0.96141
8	R-Square (COD)	0.92431
9	Adj. R-Square	0.91927

EB

	A	B
1	Equation	y = a + b*x
2	Plot	ln(σT\+(1/2))
3	Weight	No Weighting
4	Intercept	7.46047 ± 0.07647
5	Slope	-0.35797 ± 0.00594
6	Residual Sum of Squares	0.30345
7	Pearson's r	-0.9972
8	R-Square (COD)	0.9944
9	Adj. R-Square	0.99403

ETFB

	A	B
1	Equation	y = a + b*x
2	Plot	ln(σT\+(1/2))
3	Weight	No Weighting
4	Intercept	0.63175 ± 0.05465
5	Slope	-0.16834 ± 0.00495
6	Residual Sum of Squares	0.15498
7	Pearson's r	-0.99356
8	R-Square (COD)	0.98716
9	Adj. R-Square	0.98631

EHFB

Equation	y = a + b*x
Plot	ln(σT\+(1/2))
Weight	No Weighting
Intercept	0.09915 ± 0.089
Slope	-0.11822 ± 0.00
Residual Sum of Sq	0.42
Pearson's r	-0.96608
R-Square (COD)	0.93331
Adj. R-Square	0.92886

TFEB

	A	B
1	Equation	y = a + b*x
2	Plot	ln(σT\+(1/2))
3	Weight	No Weighting
4	Intercept	0.44846 ± 0.11784
5	Slope	-0.16972 ± 0.01069
6	Residual Sum of Squares	0.72061
7	Pearson's r	-0.97152
8	R-Square (COD)	0.94385
9	Adj. R-Square	0.94011

Supplementary Fig. 6| a-e, The fitting results for the four different electrolytes: (a) Base, (b) EB, (c) ETFB, (d) EHFB, (e) TFEB. For the Base electrolyte, the data were fitted by the Arrhenius

equation (equation1), because of the solidification of the electrolyte under −50 °C. The other three electrolytes were fitted by the Vogel-Tamman-Fulcher (VTF) empirical equation (equation 2). f, the optical image of conductivity test of electrolytes. g, The corresponding fitting parameters.

$$\sigma = \sigma_0 \exp(\frac{-E_a}{RT}) \qquad (1)$$

$$\sigma = \sigma_0 T^{-1/2} \exp(\frac{-Ea}{R(T-T_0)}) \qquad (2)$$

where E_a is the activation energy, σ_0 is the pre-exponential factor, T_0 is the ideal glass transition temperature, and R is the gas constant.

Supplementary Table 3: Conductivity of various electrolytes under different temperatures.

Electrolyte	σ at 25 °C (mS/cm)	σ at −80 °C (mS/cm)	σ at −90 °C (mS/cm)	E_a (eV)
Base	10.03	0.00676	0.00107	19.19
EB	11	0.191	0.0399	2.98
ETFB	10.65	0.83	0.264	1.40
EHFB	8.922	1.675	1.462	0.98
TFEB	9.525	1.083	0.564	1.41
1.28 M LiFSI FEMC/FEC- D2[1]	2.3	0.011	-	0.034

Stokes-Einstein Eqation: $$q = \frac{|ZiZj|e^2}{8\pi\varepsilon_0\varepsilon K_B T} \qquad (3)$$

Where, q is the charge of solute, Z is the valence of ion, e is charge of an electron, 1.602×10^{-19} C, ε_0 is the relative permittivity (F/m), ε is permittivity, K_B is Boltzmann constant, T is temperature.

Diffusion in liquid: $$D = \frac{K_B T}{6\pi\eta r} \qquad (4)$$

Where, D is the diffusivity of solute, r is the inter-particle distance, η is viscosity.

Diffusivity vs. ionic conductivity: $$\sigma = \frac{q^2 C D}{K_B T} \qquad (5)$$

Where, σ is ion conductivity, C is the concentration.

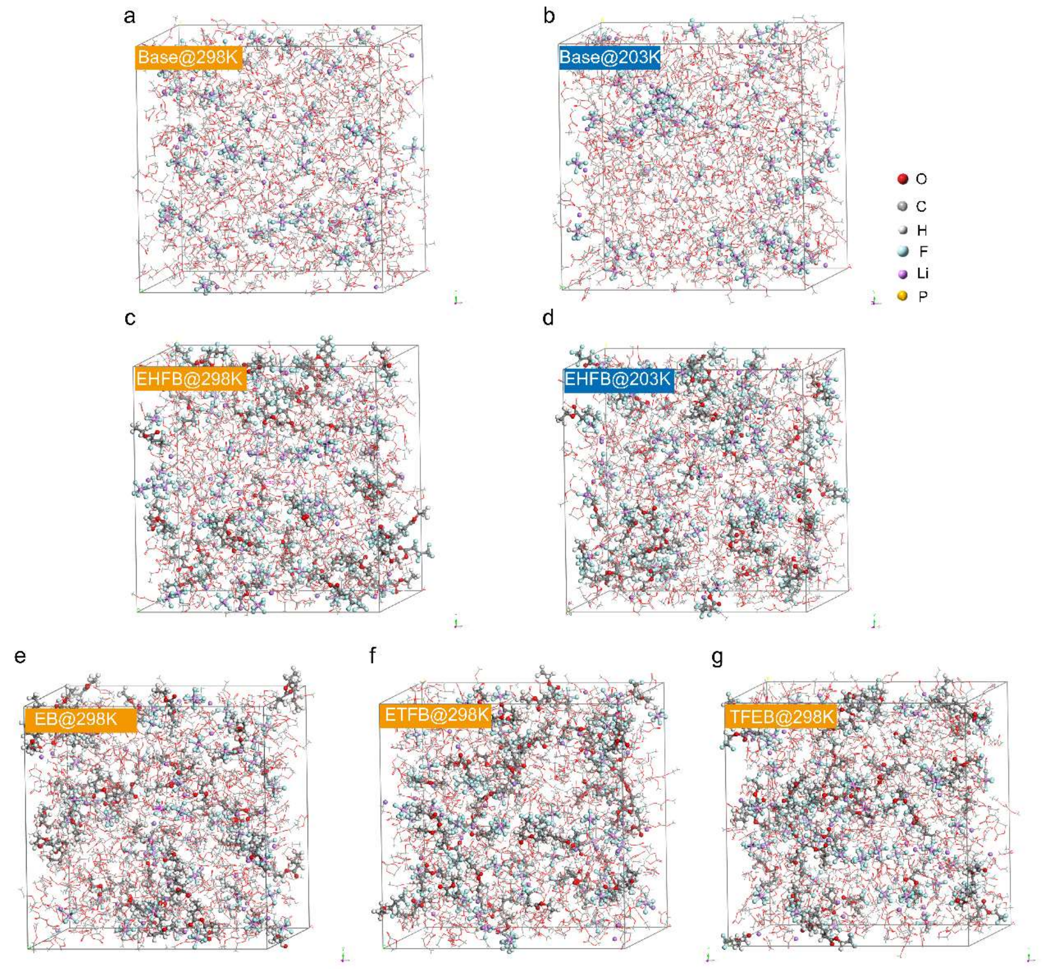

Supplementary Fig.7| Snapshots of Base (a-b), EB (e), ETFB (f), EHFB (c-d) and TFEB (g) electrolytes obtained by MD simulation at 298 K (a, c, e, f, g) and 203 K (b, d). The Li^+ and their primary coordinated shells (within 2.6 Å of Li^+ ions) are presented by ball and stick models, while the wireframes stand for free solvents.

Supplementary Table 4: Moles of different solvents in the five electrolyte systems.

solvent	Density (g/cm^3)	Volume (mL)	Relative molecular mass (g/mol)	moles
EC	1.3214	2	88.06	0.030011356
PC	1.198	1	103.09	0.011620914
DEC(Base)	0.975	3	118.1	0.024767146
DEC(Exp)	0.975	1.5	118.1	0.012383573
EMC	1.007	4	104.1	0.038693564
EB	0.9	1.5	116.158	0.011622101
ETFB	1.16	1.5	170.13	0.010227473
EHFB	1.396	1.5	242.092	0.008649604
TFEB	1.127	1.5	170.13	0.009936519

Supplementary Table 5: The numbers of different molecules in the five systems.

	Category	$LiPF_6$	EC	PC	DEC	EMC	Co-solvent	Co-efficient	Total Number	Optimized Volume($Å^3$)
Bas	Moles	0.01	0.03	0.01	0.02	0.04	—	1000	—	—

e	Number	50	150	58	124	193	—	—	575	86348.5
	Density(ρ)	$5.79e^{-4}$	$1.73e^{-3}$	$6.73\ e^{-4}$	$1.43e^{-3}$	$2.24\ e^{-3}$	—	—	$6.66e^{-3}$	—
EB	Moles	0.01	0.03	0.01	0.012	0.04	0.012	1000	—	—
	Number	50	150	58	62	193	58	—	572	84444.7
	Density(ρ)	$5.92e^{-4}$	$1.18e^{-3}$	$6.88\ e^{-4}$	$7.33e^{-4}$	$2.29e^{-3}$	$6.88e^{-4}$	—	$6.77e^{-3}$	—
ETFB	Moles	0.01	0.03	0.01	0.012	0.04	0.01	1000	—	—
	Number	50	150	58	62	193	51	—	565	85312.6
	Density(ρ)	$5.86e^{-4}$	$1.76e^{-3}$	$6.81\ e^{-4}$	$7.26e^{-4}$	$2.27e^{-3}$	$5.99e^{-4}$	—	$6.62e^{-3}$	—
EHFB	Moles	0.01	0.03	0.01	0.012	0.04	0.012	1000	—	—
	Number	50	150	58	62	193	58	—	557	83505.9
	Density(ρ)	$5.99e^{-4}$	$1.80e^{-3}$	$6.96\ e^{-4}$	$7.41e^{-4}$	$2.32e^{-3}$	$5.18e^{-4}$	—	$6.67e^{-3}$	—
TFEB	Moles	0.01	0.03	0.01	0.012	0.04	0.012	1000	—	—
	Number	50	150	58	62	193	58	—	563	84828.3
	Density(ρ)	$5.89e^{-4}$	$1.77e^{-3}$	$6.85e^{-4}$	$7.30e^{-4}$	$2.28e^{-3}$	$5.86e^{-4}$	—	$6.64e^{-3}$	—

$$n(r') = 4\pi\rho \int_0^{r'} g(r) r^2 dr \qquad (6)$$

Coordination number is calculated from equation 6 based on the radial distribution function (RDF) according to molecular dynamic simulation. Where, r′ is the first solvation radius, ρ is the number density which equals the number (N) divided by the volume (V), g(r) is the radial distribution function.

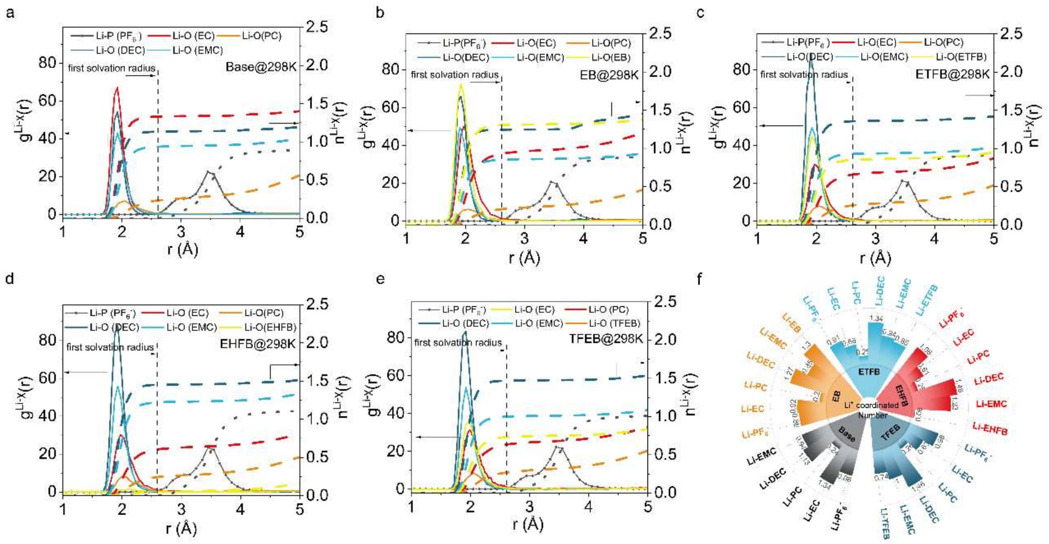

Supplementary Fig.8| Radial distribution functions ((g(r)), solid lines) and coordination numbers ((n(r)), dash lines) under 298K for Base electrolyte (a), EB electrolyte (b), ETFB electrolyte (c), EHFB electrolyte (d) and TFEB electrolyte (e), The summarized coordinated number of Li^+ with various solvents (EC, PC, DEC, EMC, EB, ETFB, EHFB, and TFEB) in the formula. (f), the summarized coordination numbers for the five studied electrolytes at 25 °C based on the calculated RDFs.

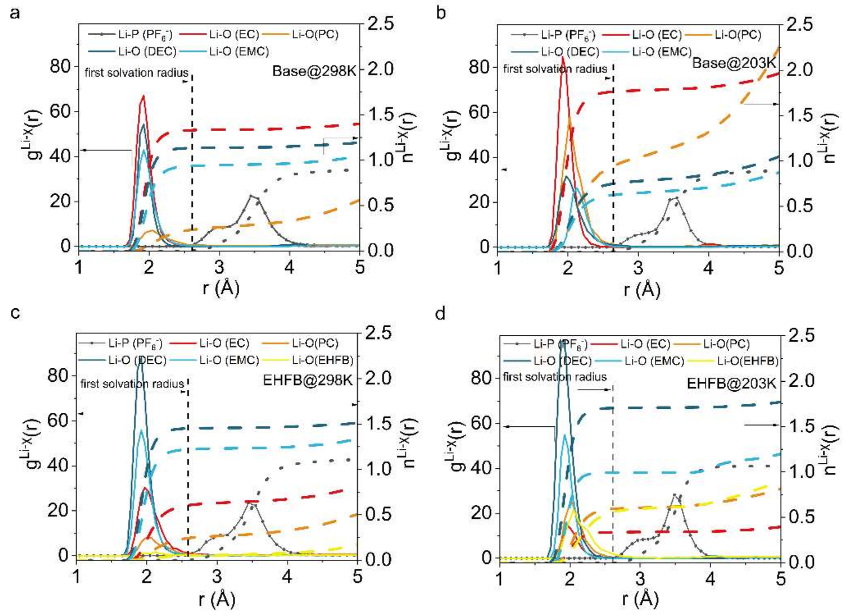

Supplementary Fig.9| Radial distribution functions (g(r)) and coordination numbers (n(r)) for Base electrolyte (a-b) and EHFB electrolyte (c-d) under 298K (a, c) and 203K (b. d) respectively.

Supplementary Table 6: Maxima (r_{max}) of the first solvation shell, radial distribution functions $g(r_{max})$, and the coordination number $N(r_{min})$ of different electrolytes.

Electrolyte	Pair	T/K	r_{max} / Å	g(max)	$N(r_{min})$
Base	Li-O_{EC}	298K	2.62	67.02	1.34
	Li-O_{PC}		2.60	7.13	0.24
	Li-O_{DEC}		2.45	54.30	1.13
	Li-O_{EMC}		2.61	42.90	0.94
	Li-P		4.20	22.42	0.86
Base	Li-O_{EC}	203K	2.52	84.76	1.76
	Li-O_{PC}		2.62	58.07	0.94
	Li-O_{DEC}		2.60	31.56	0.74
	Li-O_{EMC}		2.62	26.43	0.63
	Li-P		4.35	21.97	0.86
EB	Li-O_{EC}	298K	2.65	49.99	0.92
	Li-O_{PC}		2.63	6.20	0.2
	Li-O_{DEC}		2.52	66.02	1.27
	Li-O_{EMC}		2.53	49.23	0.85
	Li- O_{EB}		2.62	72.48	1.3
	Li-P		4.15	20.64	0.86
ETFB	Li-O_{EC}	298K	2.65	30.00	0.66
	Li-O_{PC}		2.62	8.09	0.25
	Li-O_{DEC}		2.57	88.98	1.34
	Li-O_{EMC}		2.57	49.53	0.94
	Li- O_{ETFB}		2.55	41.69	0.85
	Li-P		4.15	21.33	0.91
EHFB	Li-O_{EC}	298K	2.69	30.31	0.61
	Li-O_{PC}		2.65	7.72	0.25
	Li-O_{DEC}		2.57	88.32	1.46
	Li-O_{EMC}		2.57	55.89	1.23
	Li- O_{EHFB}		2.60	1.63	0.06
	Li-P		4.15	23.43	1.08
EHFB	Li-O_{EC}	203K	2.62	14.49	0.33
	Li-O_{PC}		2.65	25.33	0.58
	Li-O_{DEC}		2.57	96.77	1.71
	Li-O_{EMC}		2.57	54.92	0.99
	Li- O_{EHFB}		2.70	21.49	0.56

	Li-P		4.10	28.20	1.06
TFEB	Li-O$_{EC}$	298K	2.62	31.34	0.63
	Li-O$_{PC}$		2.65	8.60	0.26
	Li-O$_{DEC}$		2.57	83.38	1.46
	Li-O$_{EMC}$		2.57	53.79	1
	Li- O$_{TFEB}$		2.60	36.02	0.74
	Li-P		4.12	22.39	0.98

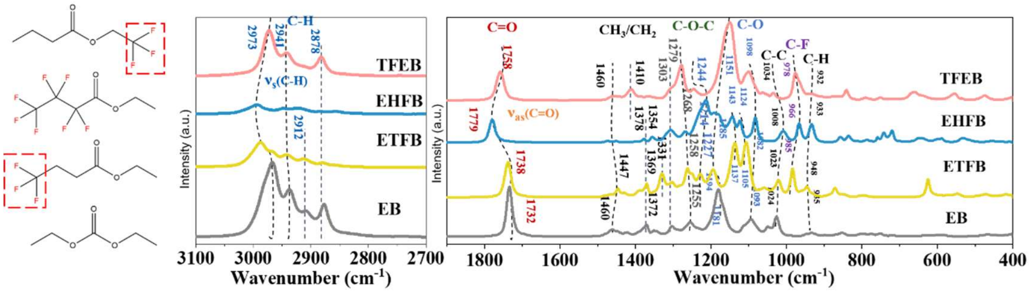

Supplementary Fig.10| FTIR spectra of co-solvent molecular EB, ETFB, EHFB, and TFEB, the peak assignment has been presented in Supplementary Table 7.

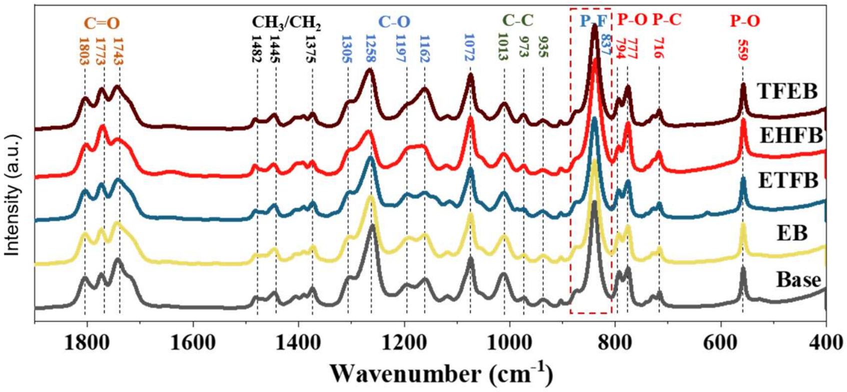

Supplementary Fig.11| FTIR spectra of different electrolytes, the peak assignment has been presented in Supplementary Table 7.

The relative abundance of free and solvated EC and DEC in different electrolyte solutions can be estimated from the stretching frequencies of their IR-active C=O groups, which occur in the region of 1660-1870 cm^{-1} and are sensitive to changes in the Li$^+$ solvation structure (Fig 3c). To evaluate the effect of different cosolvents on the solvation structure and quantify the relative abundance of solvated EC and DEC, the free and solvated C=O peaks were fitted using the Voigt functions (Fig 3d, Fig S12-S16). More details about the peaks assignment can be found in Supplementary Table 7 and Supplementary Table 8, and two parameters, R1 (the ratios of solvated to free

EC) and R2 (solvated EC to solvated DEC), were introduced using Supplementary Equation 1 and Equation 2 to quantify the relative abundance of solvated EC and DEC as well as the evolution of competence degree for coordination sites between EC and DEC. The FTIR spectrum of the EHFB electrolyte has five main peaks – two centered at approximately 1724 and 1746cm^{-1} that were assigned to DEC, and three at 1772, 1803, 1836 cm^{-1} that were assigned to EC. The high-frequency peaks at 1746 and 1803 cm^{-1} were assigned to the C=O stretching modes of free DEC and EC, respectively, while the low frequency bands at 1724 and 1772 cm^{-1} were assigned to Li^+ coordinated DEC and EC[2, 3, 4], respectively, peak at 1836 cm^{-1} was observed only at low temperature and was assigned to the ring lactone vibration of EC. The vibrational and fitting parameters for these spectra are given in Supplementary Table 7 and Supplementary Table 8, respectively.

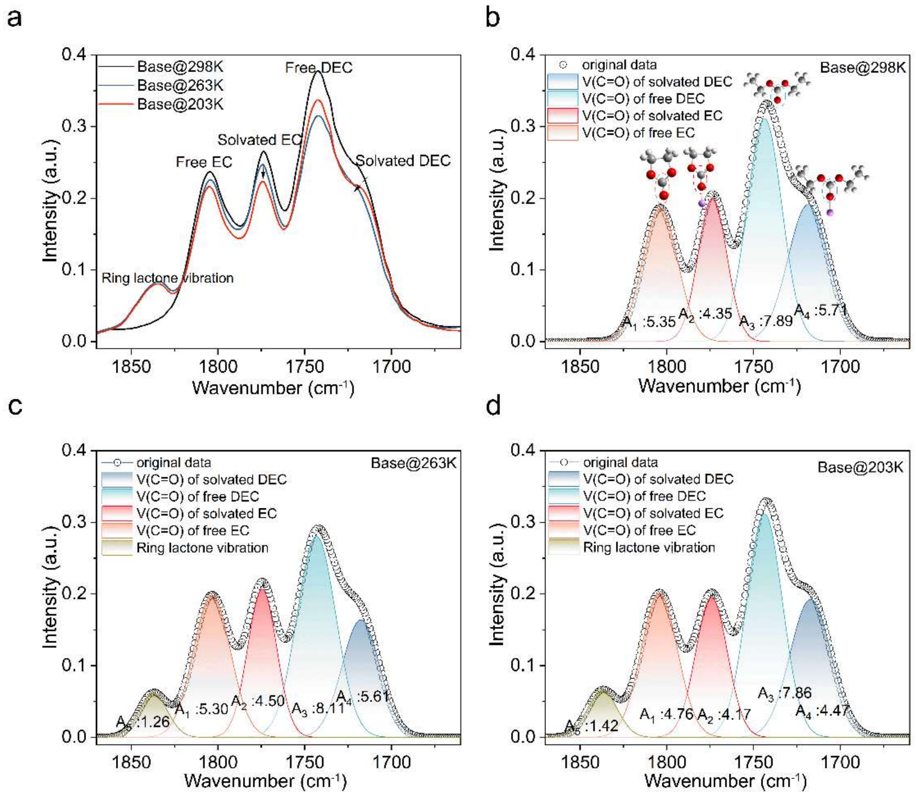

Supplementary Fig.12| FTIR spectra of EC and DEC in Base electrolyte. a, Comparison of the C=O vibration modes under different temperature; b-d, Fitting spectra with the Voigt function in 298K, 263K, and 203K, respectively.

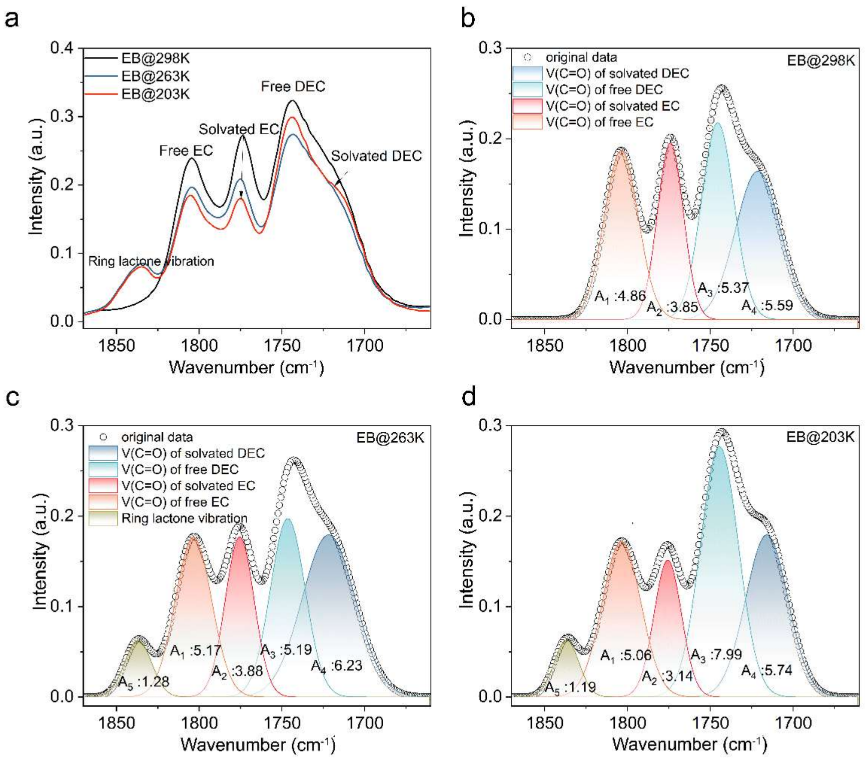

Supplementary Fig.13| FTIR spectra of EC and DEC in EB electrolyte. a, Comparison of the C=O vibration modes under different temperature; b-d, Fitting spectra with the Voigt function in 298K, 263K, and 203K, respectively.

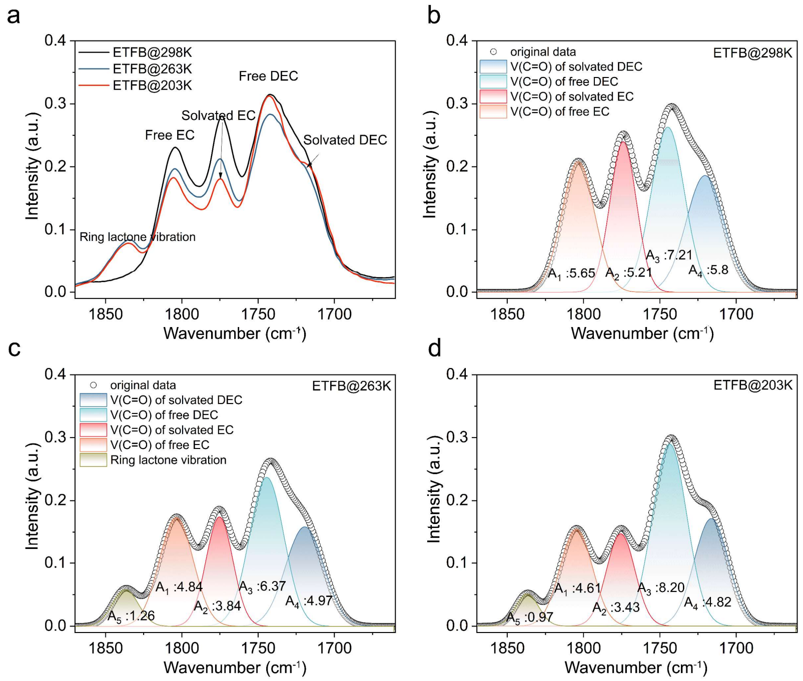

Supplementary Fig.14| FTIR spectra of EC and DEC in ETFB electrolyte. a, Comparison of the C=O vibration modes under different temperature; b-d, Fitting spectra with the Voigt function in 298K, 263K, and 203K, respectively.

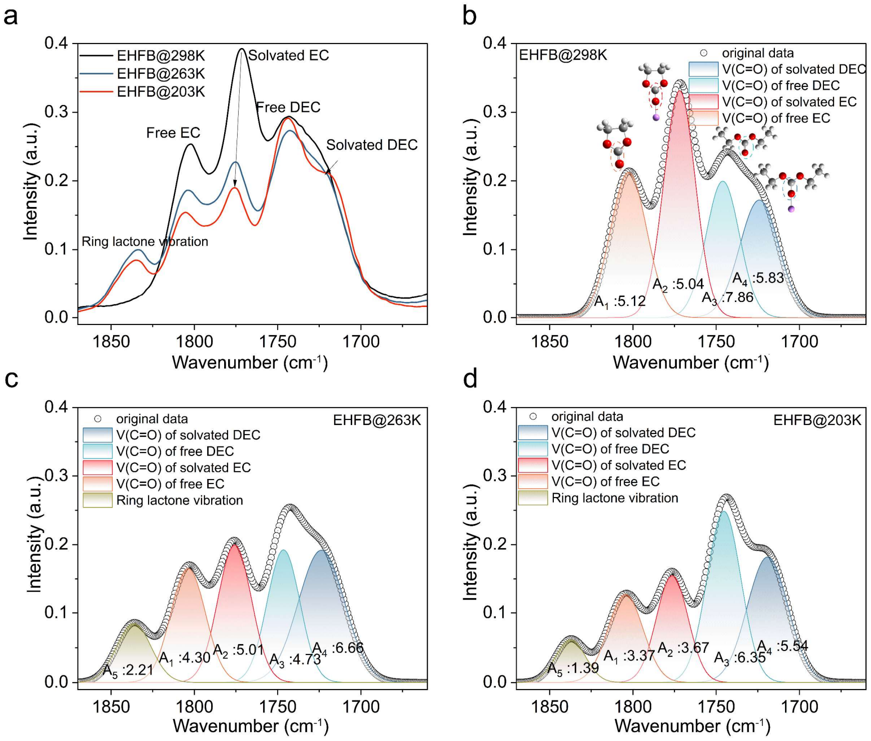

Supplementary Fig.15| FTIR spectra of EC and DEC in EHFB electrolyte. a, Comparison of the C=O vibration modes under different temperature; b-d, Fitting spectra with the Voigt function in 298K, 263K, and 203K, respectively.

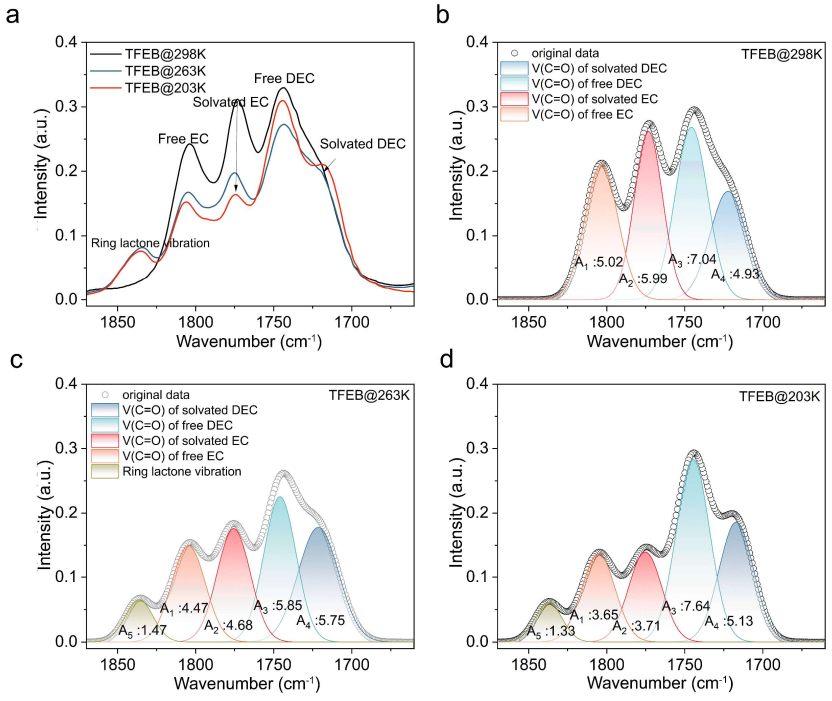

Supplementary Fig.16| FTIR spectra of EC and DEC in TFEB electrolyte. a, Comparison of the C=O vibration modes under different temperature; b-d, Fitting spectra with the Voigt function in 298K, 263K, and 203K, respectively.

Supplementary Table 7 The FT-IR peak information of co-solvent contained electrolyte and co-solvents.

Number	Wavenumber (cm^{-1})					Band Assignment	Description	References
	Electrolytes	EB	ETFB	EHFB	TFEB			
1	2989	2967	2987	2993(weak)	2973	V_s(C-H) of β site	Moving towards high wavenumber after solvation	[5, 6]
2	2938	2937	2941 (weak)	None	2941	V_s(C-H) of $-CH_2-$		[5]
3	2913(weak)	2908	2912 (weak)	None	None	V(-H)		[6]
4	2878 (weak)	2878	2878 (weak)	None	2881	V_s(C-H) of $-CH_3$		[5, 7]
5	1743	1732	1738	1779	1758	V(C=O)/ or V(C=O) of DEC	Affected by the electron-withdrawing group F, the C = O stretching vibration frequency shifts towards higher frequencies due to the inductive effect.	[2, 6, 8]
6	1773	—	—	—	—	V(C=O) of solvated EC		[2, 3, 7, 8]
7	1803	—	—	—	—	V(C=O) of free EC		[2, 3, 7, 8]
8	—	1460(weak)	1447	—	1460	$\sigma_{as}(CH_3)$ of solvent		[3, 6, 8, 9]
9	1445	—	—	—	—	$\sigma_{as}(CH_3)$	Moving towards low wavenumber after solvation	[3, 8]
10	1482	—	—	—	—	σ (CH_2)		[2, 9]
11	—	—	—	—	1410	σ (CH_2)		[2, 3, 8, 9]
12	1375	1372	1369	1378(weak)	—	σ (CH_3), characteristic peak		[2]
13	—	—	1331	—	—	V(O=C-O) of ETFB		[3, 8]
14	1305(shoulder)	1303	1303	1306	1305			[2, 8]
15	1258	1255	1258	1268	1279	V(C-O) of carboxylic acid	p-π Conjugation Increases the Density of Electron Clouds between C and O	[2, 3, 9]
16	—	—	1227	1214	1244	V_{as}(C-O-C)		[2]
17	1197	1181	1194\1137	1185\1143\ 1124	1151	High wavenumber V(C-O) of Co-solvent Low wavenumber V(C-O) of solvated		[2, 3, 8]
18	1162	—	—	—	—	V(C-O) of free solvent		[2, 3, 8]
19	1072	1093	1105	1082	1098	V(C-O) of free solvent		[8, 10]
20	—	1052(weak)	1059(weak)	—	—	V(C-O) of co-solvent		[2]

21	1013	1024	1023	1008	1034(weak)	V(C-C) of DEC		[2]
22	—	—	985	966	978	V(C-C) of co-solvent		
23	973(weak)	—	—	—	—	V(C-C) of EC		[2]
24	935(weak)	935(weak)	948	933	932(weak)	C-H	External deformation vibration	[5]
25	902	—	—	—	—			
26	—	854(weak)	870	857\835	841	Impurity of co-solvent		
27	837	—	—	—	—	V(P-F)		[3, 10]
28	873	—	—	—	—	$V(Li^+-PF_6^-)/V(Li^+-PF_6^-)_2$		[10]
29	—	—	—	742	—	$\sigma (COO^-)$		
30	794	—	—	—	—	V(P-O)		
31	777	—	—	—	—	V(P-O)		
32	716	—	—	—	—	V(P-C)		
33	559	—	—	—	—			
34	—	—	626	647(weak)	663	Impurity of co-solvent		
35	—	—	—	586	555	Impurity of co-solvent		
36	—	—	—	531	475	Impurity of co-solvent		

Supplementary Table 8: Fitting parameters of the C=O stretching modes in the experimental FTIR Spectra.

Sample	T(K)	Peak position					FWHM					Peak Aera					Aera Percentage				
		A_1	A_2	A_3	A_4	A_5	A_1	A_2	A_3	A_4	A_5	A_1	A_2	A_3	A_4	A_5	A_1	A_2	A_3	A_4	A_5
Base	298K	1803.5	1774.5	1743.2	1717.1		25.3	19.77	26.43	27.9		5.35	4.35	7.89	5.71		22.95	18.64	33.83	22.81	
	263K	1804.33	1774.21	1743.69	1718.47	1837.32	24.79	21.65	24.42	27.58	19.36	5.3	4.5	8.11	5.61	1.26	21.2	17.95	32.38	25.05	5.42
	203K	1803.9	1773.69	1743.71	1717.64	1837.09	23.94	19.61	23.73	25.6	20.39	4.76	4.18	7.86	4.47	1.42	21.24	18.63	35.08	19.15	5.66
EB	298K	1803.79	1774.07	1745.51	1721.22		24.39	18.5	23.16	31.99		4.86	3.85	5.37	5.59		24.71	19.57	27.31	28.42	
	263K	1803.45	1775.52	1746.46	1721.77	1836.9	25.93	20.63	24.74	37.88	19.3	5.17	3.88	5.19	6.23	1.28	21.71	17.29	23.17	32.18	5.71
	203K	1803.61	1775.67	1744.48	1715.87	1836.83	27.6	19.54	27.13	30.07	17.79	5.06	3.14	7.99	5.74	1.19	21.89	13.6	34.58	24.8	5.13
ETFB	298K	1803.55	1774.03	1744.79	1720.38		25.49	20.4	25.77	29.28		5.65	5.21	7.21	5.8		23.68	21.81	30.22	24.29	
	263K	1803.6	1775.33	1744.32	1719.41	1837.05	26.19	20.66	26.64	29.39	20.21	4.84	3.84	6.37	4.97	1.26	22.38	17.74	31.11	22.95	5.82
	203K	1804.79	1775.65	1743.57	1716.26	1837.01	24.93	21.87	26.59	26.43	17.82	4.61	3.43	8.2	4.82	0.97	19.1	15.92	38.08	22.39	4.5
EHFB	298K	1802.57	1771.94	1746.01	1724.07		25.75	22.27	23.73	27.95		5.12	5.04	7.86	5.83		24.43	32.94	21.15	21.48	
	263K	1803.73	1775.75	1746.43	1723.68	1836.34	24.44	23.62	23.12	32.62	24.48	4.3	5.01	4.73	6.66	2.21	18.76	21.88	20.65	29.06	9.64
	203K	1804.47	1776.36	1745.33	1719.08	1837.42	25.03	22.3	24.01	28.64	21.15	3.37	3.67	6.35	5.54	1.39	16.61	18.06	31.23	27.26	6.84
TFEB	298K	1803.4	1773.22	1745.71	1722.2		24.97	21.5	24.68	27.4		5.02	5.99	7.04	4.93		23.85	25.39	29.87	20.88	
	263K	1804.61	1775.59	1745.95	1721.37	1836.2	25.54	24.99	24.38	30.38	21.12	4.47	4.68	5.85	5.75	1.47	18.64	21.46	26.79	26.37	6.74
	203K	1805.3	1775.32	1744.73	1716.95	1837.31	25.09	25.02	25.37	25.93	20.44	3.65	3.71	7.64	5.13	1.33	16.99	17.31	35.61	23.9	6.18

Supplementary Table 9: Principal SEI products formed on graphite electrode

Fragment ion	Corresponding SEI products	Category	Formation/ Origin	Reference
CH/CH_2	$R\text{-}CH(CH_2)$	Organic SEI	Organic Products contained CH/CH_2 group	
CO_3	$ROCO_2Li$		Eq1: $2EC+2e^{-}+2Li^{+}\rightarrow(CH_2OCO_2Li)_2+CH_2=CH_2$<br>Eq2: $EC+2e^{-}+2Li^{+}\rightarrow LiCH_2CH_2OCO_2Li$	11, 12, 13, 14, 15, 16, 17
			Eq3: $EC+e^{-}+Li^{+}\rightarrow CH_2CH_2OCO_2Li$<br>Eq4: $2EC+2e^{-}+2Li^{+}\rightarrow(CH_2CH_2OCO_2Li)_2$<br>Eq5: $2EC+2e^{-}+2Li^{+}\rightarrow LiOCO_2\,(CH_2)_4OCO_2Li$<br>Eq6: $EC+2e^{-}+2Li^{+}\rightarrow Li\,(CH_2)_2OCO_2Li$	14, 18
C_2H_3O	CH_3COLi		Eq7: $DEC+e^{-}+Li^{+}\rightarrow CH_3CH_2O\cdot O+H_2CH\text{-}CH_2OLi$<br>Eq8: $DEC+e^{-}+Li^{+}\rightarrow CH_3CH_2OCO+H_2CH\text{-}CH_2OLi$	13, 18
$C_2H_3O_2$	CH_3COOLi		Eq9: $r\text{-}BL+Li\rightarrow\cdot CH_2CH_2\,CH_2\text{-}COOLi$<br>Eq10: $HCOOCH_3+e^{-}\rightarrow HCOOCH_3$<br>$HCOOCH_3\rightarrow HCOO^{-}+\cdot CH_3$<br>$HCOO^{-}+Li^{+}\rightarrow HCOOLi$<br>$\cdot CH_3\rightarrow CH_4$ or C_2H_6<br>Eq11 (Analogically obtained):<br>$C_3F_7COOC_2H_5+2Li\rightarrow RCOOLi+2CH_2+LiF$	11, 13, 18, 19
$LiF_2^{-}/Li_2F_3^{-}/Li_3F_4^{-}/Li_4F_5^{-}/$	LiF	Inorganic SEI	Eq12: $LiPF_6=PF_5+LiF$<br>Eq13: $LiPF_6+H_2O=POF_3+2HF+LiF$<br>Eq14: $POF_3+ne^{-}+nLi^{+}=LiF+Li_xPF_y$<br>Eq15: $Li_2CO_3+2HF\rightarrow 2LiF+CO_2+H_2O$	20, 21, 22, 23
$PO_3^{-}/POF_2^{-}/LiPFO_2^{-}/LiPFO_3^{-}$	Fluorophosphate and Its decomposition Products		Eq16: $POF_3+nH_2O+xLi^{+}\rightarrow Li_xPO_{1-n}F_{1-2n}+2nHF$<br>Eq17: $POF_3+H_2O=HF+HPO_2F_2$<br>Eq18: $HPO_2F_2+H_2O=HF+H_2PO_3F$<br>Eq19: $H_2PO_3F+H_2O=H_3PO_4+HF$<br>Eq20: $HPO_2F_2+Li=LiPFO_2+HF$	16, 24, 25, 26, 27, 28, 29, 30

			Eq21: $H_2PO_3F+Li^+=LiPFO_3+H^+$	
$LiCO_3^-$	Li_2CO_3		1) Two electrons reduction of EC, PC, DMC,EMC<br>Eq22: $EC+2e^-+2Li+ \rightarrow Li_2CO_3+CH_2=CH_2$<br>2) Reaction of $ROCO_2Li$ with H_2O or HF<br>Eq23: $H_2O + (CH_2OCO_2Li)_2 \rightarrow Li_2CO_3+CO_2+(CH_2OH)_2$<br>Eq24: $2CO_2+2e^-+2Li^+ \rightarrow Li_2CO_3+CO$<br>Eq25: $2RCO_3Li+H_2O \rightarrow Li_2CO_3+2ROH+ CO_2$	11, 12, 13, 14, 15, 18
LiOH	LiOH		Produced by reaction of other products with water contamination<br>Eq26: Trace $H_2O + e^-+Li^+ \rightarrow LiOH + 1/2H_2$	18
Li_2O	Li_2O		Eq27: $LiOH+ e^-+Li^+ \rightarrow Li_2O+1/2H_2$<br>Reduction of Li_2CO_3, degradation of SEI products<br>Eq28: $Li_2CO_3= Li_2O + CO_2$	18

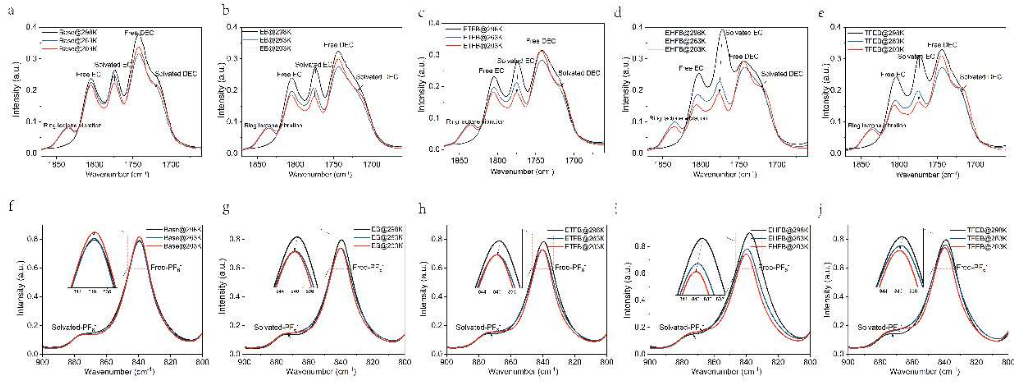

Supplementary Fig.17| FTIR spectra of C=O waveband (a-e) and P-F waveband (f-j)

It shows that the higher fluorinated cosolvent decreases the EC coordination and promotes DEC solvation to the most, as well as reduces the PF_6^- to the most.

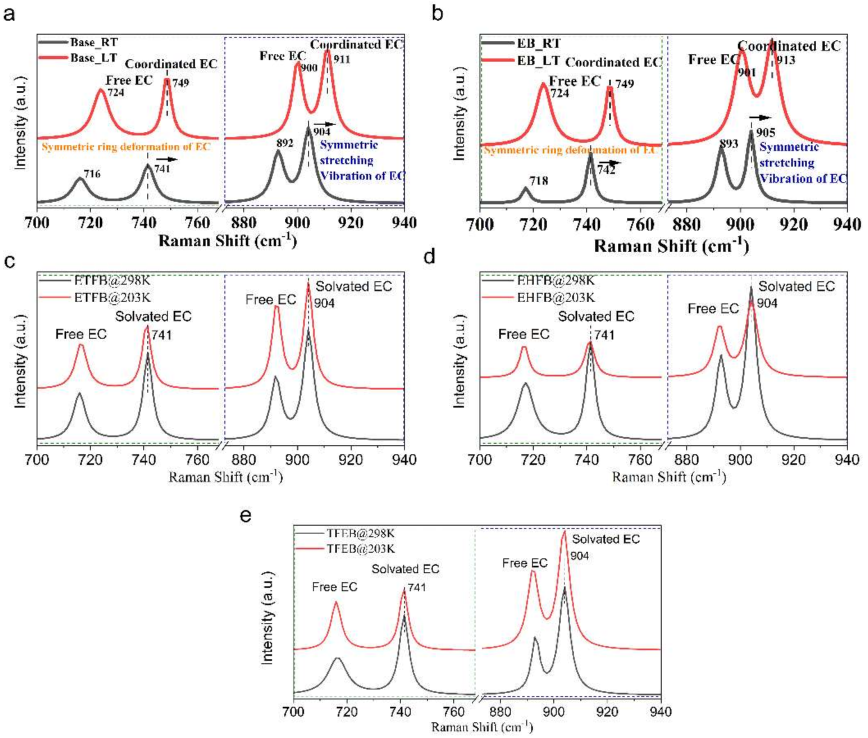

Supplementary Fig.18| Raman spectra for symmetric ring deformation of EC and stretching vibration band of C-O single bond in those five electrolytes

When introducing F in electrolyte, the blueshift will be inhibited under low T.

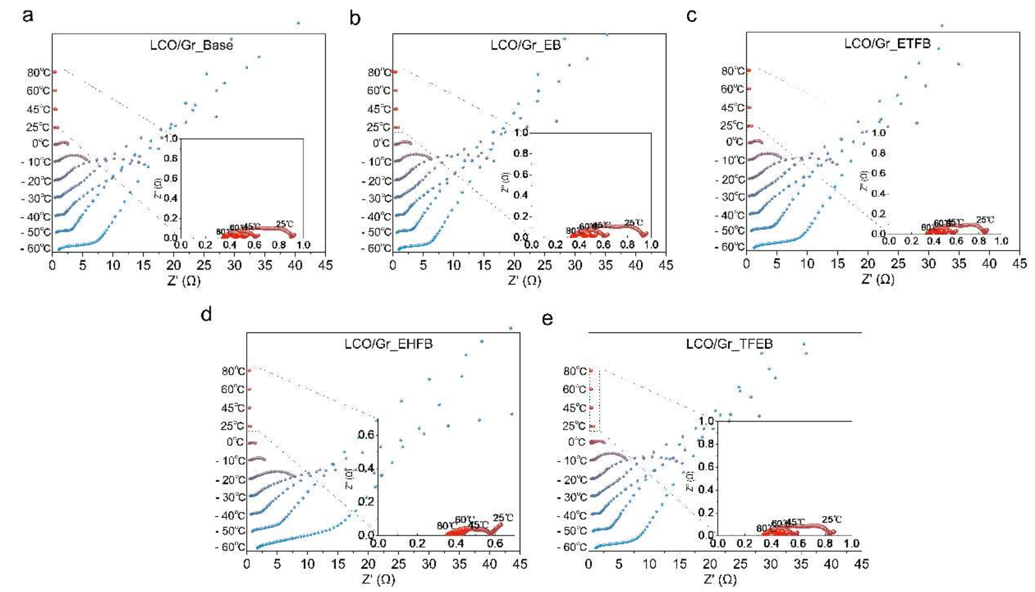

Supplementary Fig.19| Temperature-dependent electrochemical impedance spectra (EIS) in the range of 80 °C to −60 °C for 4.5V LCO/Gr pouch cells. The inset showed the EIS plot at temperatures of 25 °C to 80 °C. (a) base electrolyte, (b) EB electrolyte, (c) ETFB electrolyte, (d) EHFB electrolyte, (e) TFEB electrolyte.

The EIS of the LCO/Gr pouch cells at 50% of SOC were measured across a temperature range from 80°C to −60°C (Supplementary Fig.19). The EIS plot of LCO/Gr pouch cells consists of two plump semicircles and a line at high temperature. The high-frequency semicircle represents the impedance of Li transport in SEI and the middle-frequency semicircle is attributed to the charge transfer reaction. While, under low temperature, the EIS plot consists of an oblate semicircle and the line.

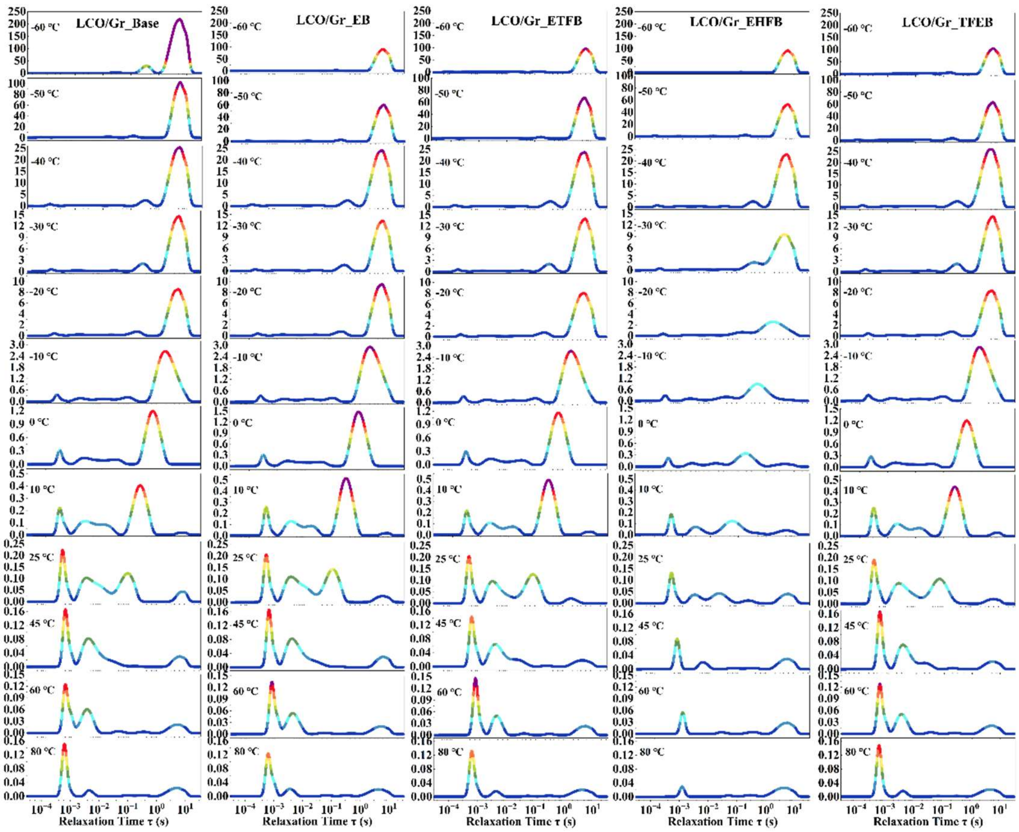

Supplementary Fig.20| DRT results at different temperature in the range of 80 °C to −60 °C for 4.5V LCO/Gr pouch cells obtained from the data in Fig S19.

Equivalent circuit models (ECM) are often used to analyze the EIS data. However, as put forward in Refs, ECM is not suitable for mechanistic analysis, as it has the polysemy problem, that is, countless ECMs can fit the same piece of EIS data, and it does not link electric elements of the ECM with physicochemical parameters of the battery. To avoid these problems associated with ECMs, distribution of relaxation times (DRT) method has been employed to quantify the contribution of each part of the resistance without artificial fitting error. Intrinsically, it is a purely mathematical transformation that translates the frequency domain Nyquist plots into the time domain DRT profile via eq 7[31, 32, 33].

$$Z_{DRT} = R_{\infty} + \int_{-\infty}^{\infty} \frac{\gamma(ln\tau)}{1+i2\pi} dln\tau \qquad (7)$$

In which each physical process is represented as a local maximum in a continuous distribution function[33]. And the area under the peak represents the impedance value[34]. To avoid the inductance characteristics in the high frequency area

(above 1KHz) derived from the effect of current collectors, therefore, EIS at the area above 1KHz cannot be calculated using the DRT method due to the long measurement time and then fitted[35]. We assigned the peak at low frequency (the τ in the range of 10^1 to 10^{-1}) as charge transfer resistance (R_{ct}), and the peak at medium frequency (the τ in the range of 10^{-1} to 10^{-3}) as the resistance of lithium-ion transport through the SEI (R_{SEI}), and the high frequency peak (10^{-3} to 10^{-4} s) is identified as contact resistance (R_0) which is in good agreement with the literature[36]. The EIS was conducted at temperature ranging from 80 °C to −60 °C at 50% SOC. R_0 is much less sensitive to the temperature, and R_{ct} and R_{SEI} regimes are all temperature-dependent, and the corresponding R_{ct} resistances increase significantly with decreasing temperature, when fluorinated co-solvent added, the resistance increment is much less.

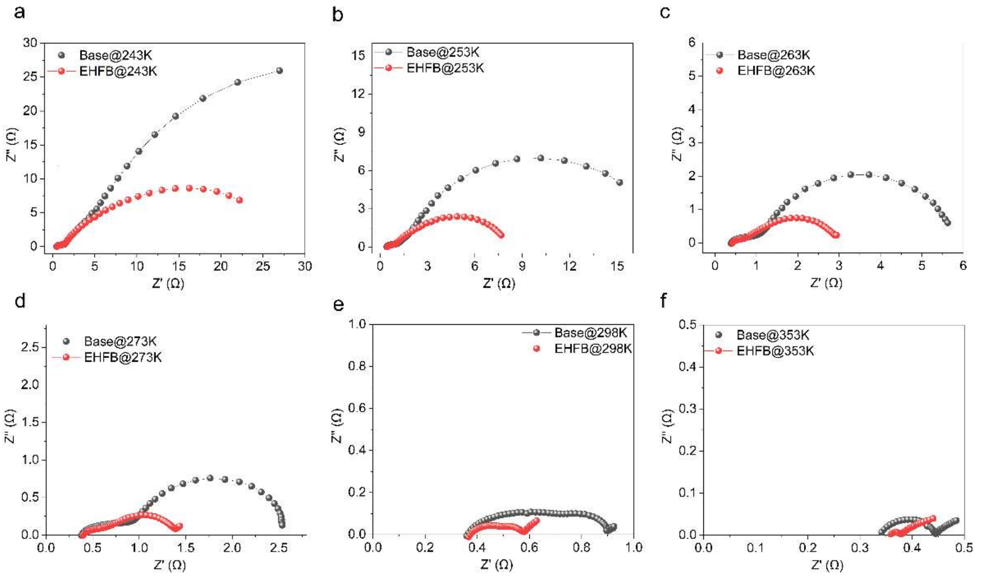

Supplementary Fig.21| EIS comparison of Base electrolyte and EHFB electrolyte under different temperature. a, 243K. b, 253K. c, 263K. d, 273K. e. 298K. f, 353K.

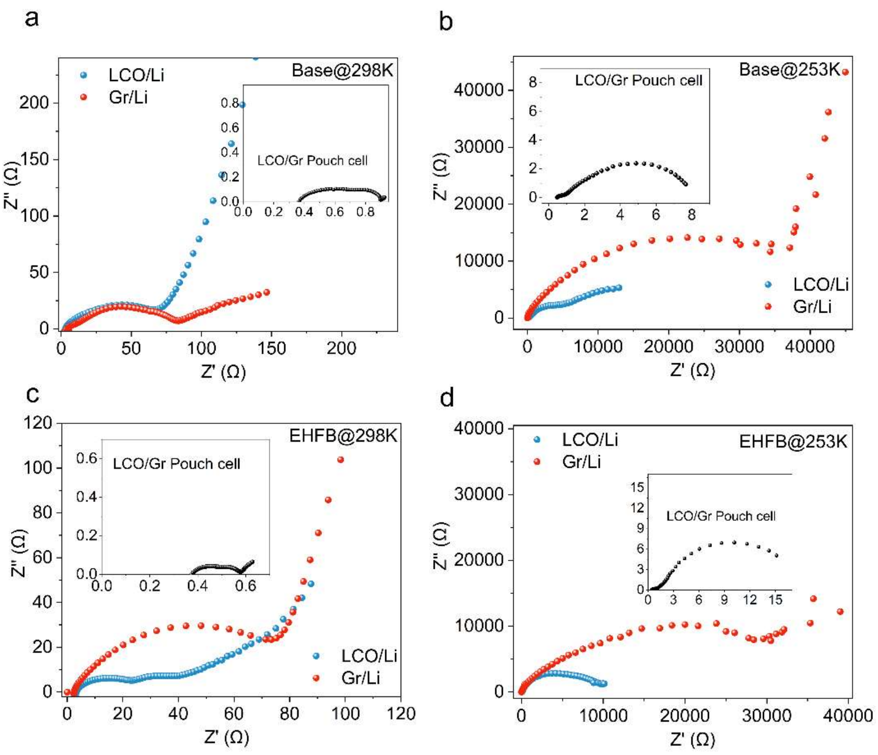

Supplementary Fig.22| EIS spectra of the LCO/Li half-cell and Gr/Li half-cell with Base electrolyte at 298K (a) and 253K (b), and with EHFB electrolyte at 298K (c) and 253K (d). The corresponding EIS spectra of LCO/Gr pouch cell is inserted.

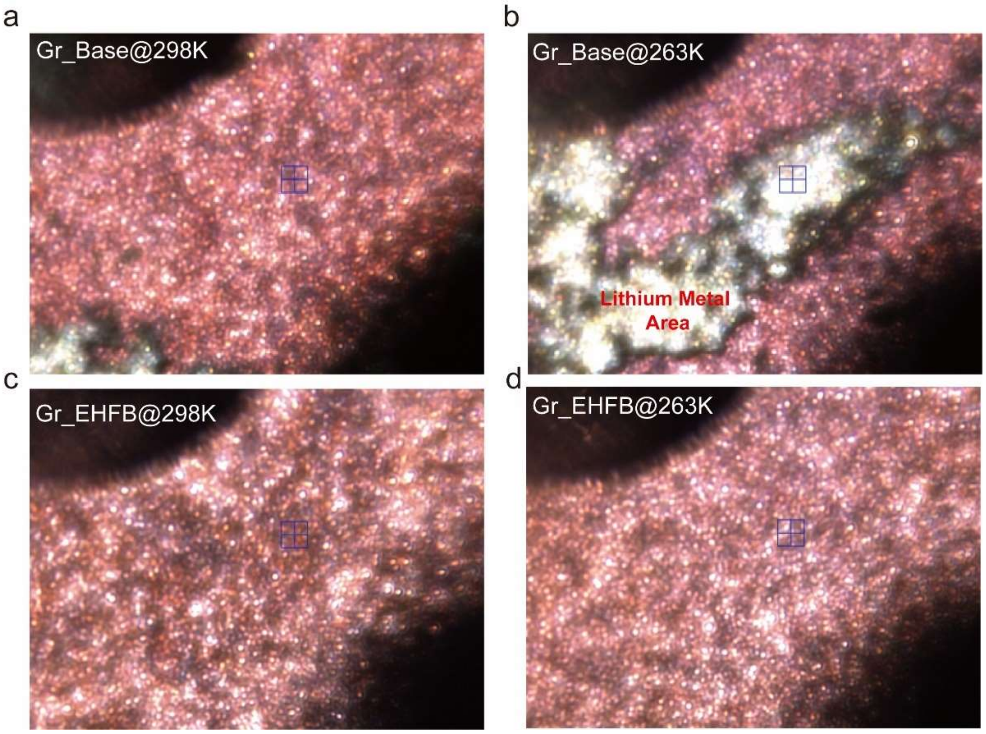

Supplementary Fig.23| Optical images seen from the TOF-SIMS camera of graphite anode cycled

in Base electrolyte (a-b) and EHFB electrolyte (c-d) at 298K and 263K, respectively.

After cycling at 263K in Base electrolyte compared with it cycled at 298K, the unevenly distributed metallic deposit (the bright area) on the graphite surface was obviously observed, which is the notorious “lithium dendrite”, while for the EHFB, there were the evenly distributed and smooth graphite intercalation compounds LiC_x even cycled at 263K. And the selected test area is located at the center of blue cursor.

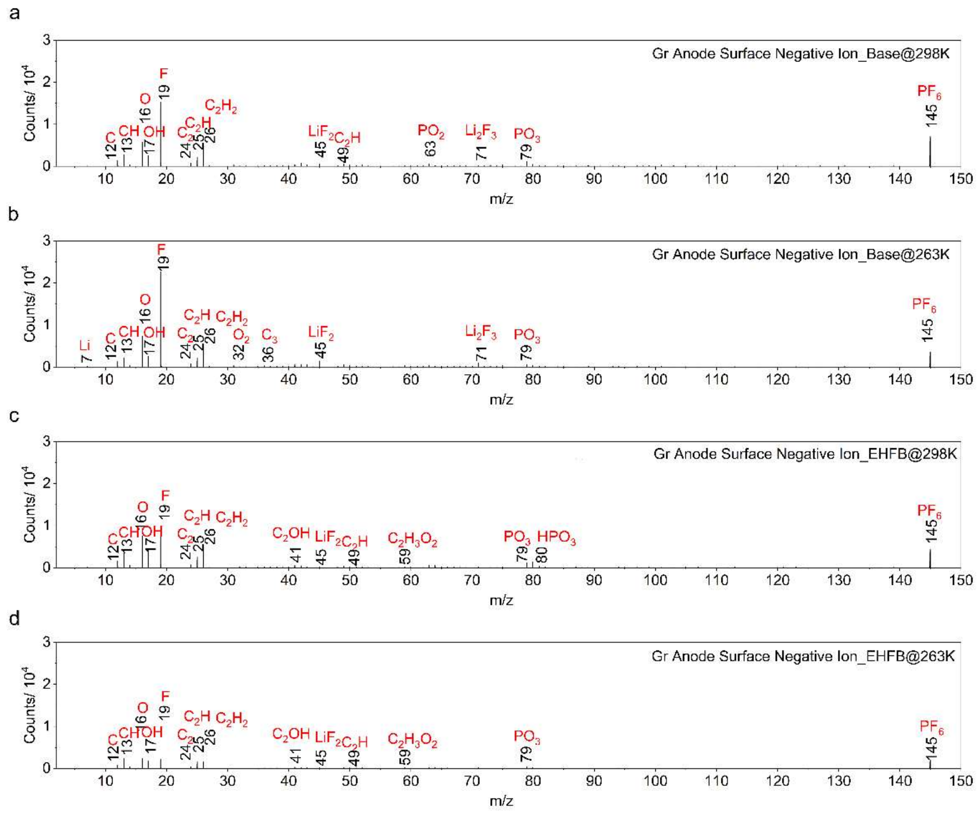

Supplementary Fig.24|The mass spectrum collected at the surface in negative ion mode of graphite electrodes cycled with the (a-b) Base electrolyte and (c-d) EHFB electrolyte after the 200th cycle at 298K and 263K.

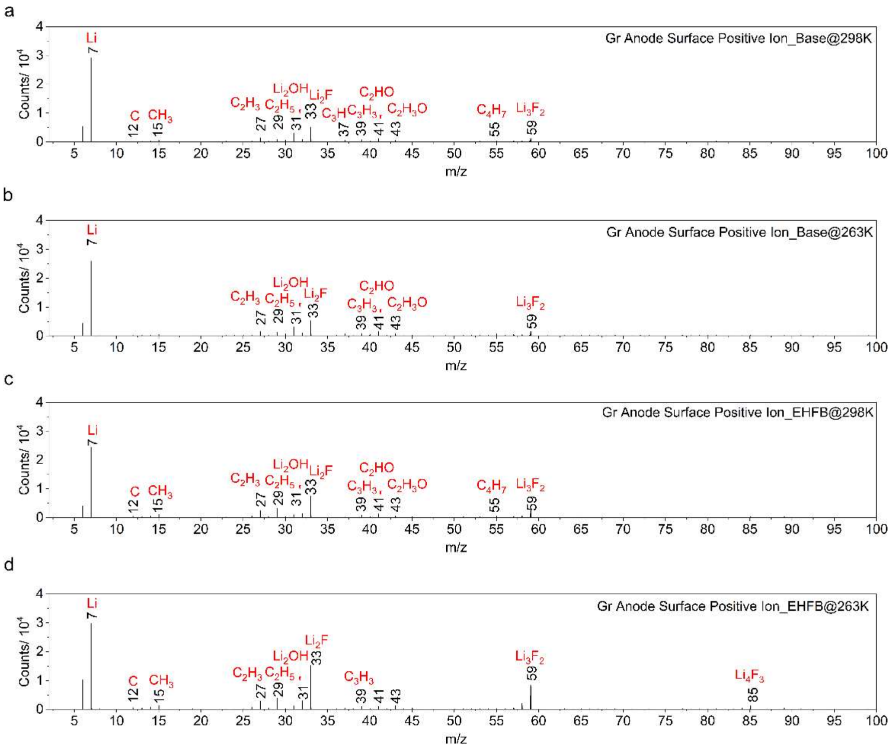

Supplementary Fig.25|The mass spectrum collected at the surface in positive ion mode of graphite electrodes cycled with the (a-b) Base electrolyte and (c-d) EHFB electrolyte after the 200th cycle at 298K and 263K.

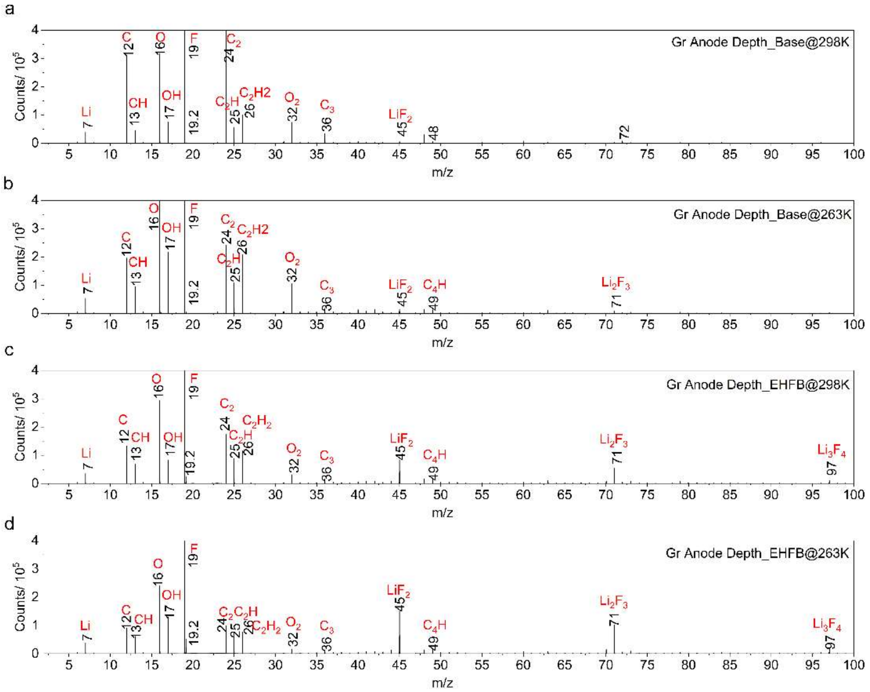

Supplementary Fig.26|The mass spectrum collected at the area after sputter in negative ion mode of graphite electrodes cycled with the (a-b) Base electrolyte and (c-d) EHFB electrolyte after the 200th cycle at 298K and 263K.

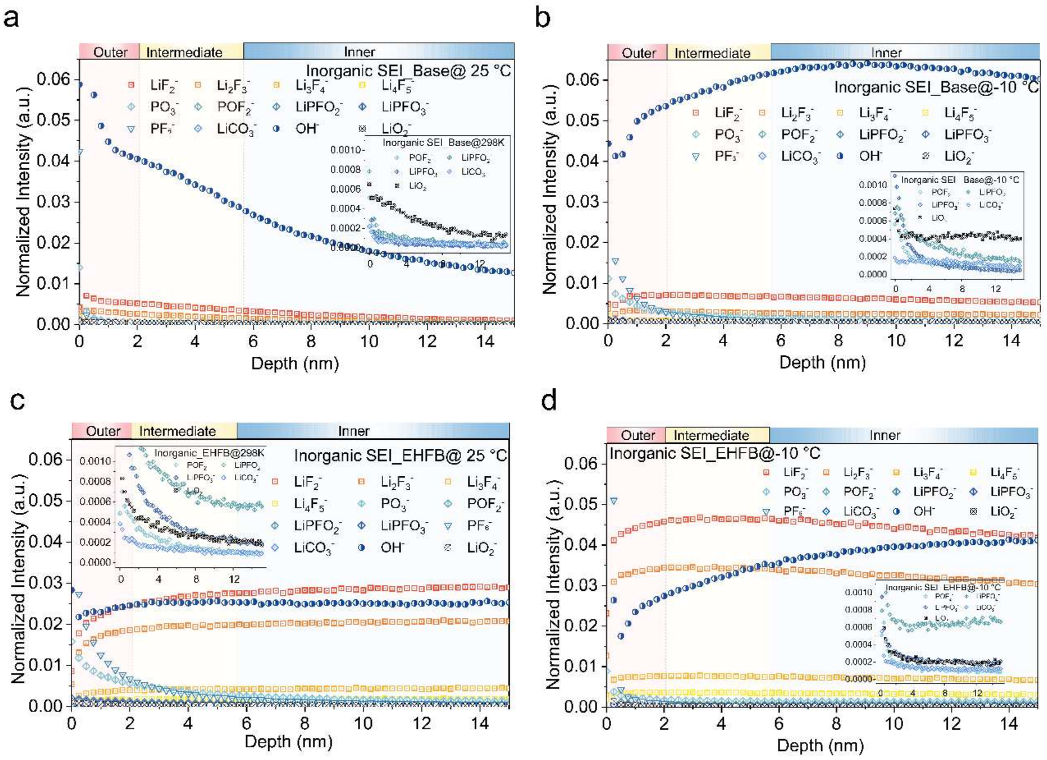

Supplementary Fig.27|The TOF-SIMS depth profiles of functional groups in inorganic SEI after long cycling for Base electrolyte (a-b) and EHFB electrolyte (c-d) at 298K and 263K.

The inorganic SEI contains LiF, $LiP_xF_yO_z$, Li_2O, and LiOH, Li_2CO_3 in all the samples. The LiF component derived from Base electrolyte (Supplementary Fig. 27a-b) is much smaller than it derived from EHFB electrolyte (Supplementary Fig. 27a-b), which is only 1/6 of that in EHFB electrolyte at low temperature (LT). Supplementary Table 7 list the origin of the main SEI products formed on graphite electrode and the possible reactions according to literatures, therefore, the less LiF means less anion to participate the solvation process and LiF has been reported repeatedly to be a good SEI component to enhance the conductive of SEI, and the LiF and LiOH component increases with the increase of depth except cycled in Base electrolyte at room temperature (RT), which is well consistent with those reported literatures that the inner layer of SEI is main consist of inorganic components, and the LiF and LiOH component produced in EHFB electrolyte at LT is enhanced compared with it at RT, which indicates that more anions participate solvation process in EHFB contained cells when temperature lowered. While, for other inorganic component Li_2CO_3, Li_2O and $LiP_xF_yO_z$ which properly originated from the two electrons reaction and the lithium salt decomposition, respectively, showed a downward trend with the depth decreases.

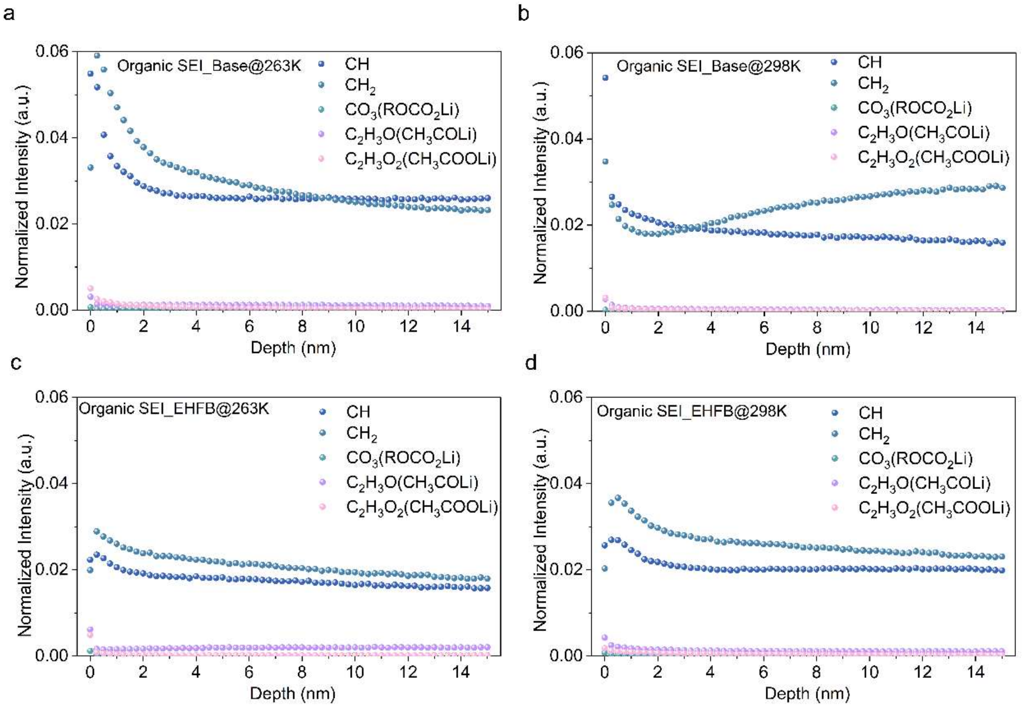

Supplementary Fig.28|The TOF-SIMS depth profiles of functional groups in organic SEI after long cycling for Base electrolyte (a-b) and EHFB electrolyte (c-d) at 298K and 263K.

For the organic components C-H and C-O-H, they all showed the downward trend in all the samples (Supplementary Fig. 28), and the general organic component originated from the decomposition of solvent in EHFB cells is smaller than in Base cells. To enlarge the difference of the three typical organic components which is covered in Supplementary Fig. 28, CO_3^-, $C_2H_3O^-$ and $C_2H_3O_2^-$ are separated to compare between Base cells and EHFB cells under LT and RT (Supplementary Fig. 29). The ion fragments CO_3^- which represent the $ROCO_2Li$ originated from the decomposition of EC molecule (Supplementary Table 7), generally showed increases with the temperature lowered in Base cells but inversely in EHFB cells, indicating that EC is promoted to solvated with Li^+ with Base electrolyte but weakened with EHFB electrolyte when temperature lowered. While for the component of $C_2H_3O^-$ which is ascribed as the CH_3COLi that derived from the decomposition of DEC (Supplementary Table 7), is totally increased and decreased when temperature is lowered in Base cells and EHFB cells, respectively, which also certificate that more DEC participates the solvation process in EHFB electrolyte than in Base electrolyte when temperature deceases. These trends of EC and DEC decomposition are rightly agreeing with the calculation result and spatial spectrum analyses.

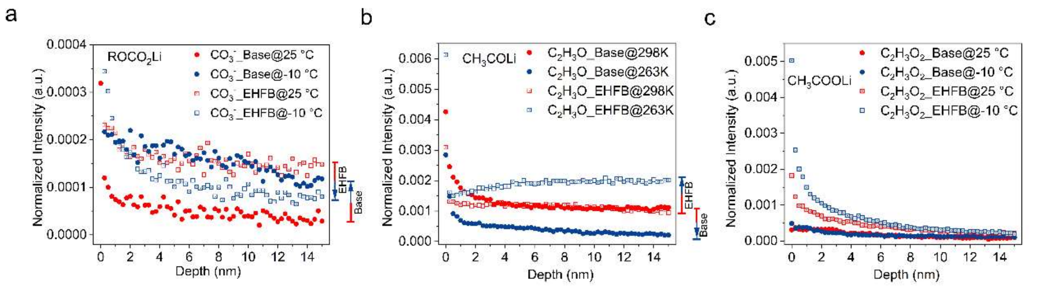

Supplementary Fig.29| The TOF-SIMS depth profiles of the three typical organic components on graphite anode cycled in Base and EHFB electrolyte at different temperature. (a) $ROCO_2Li$, (b) CH_3COLi, and (c) CH_3COOLi.

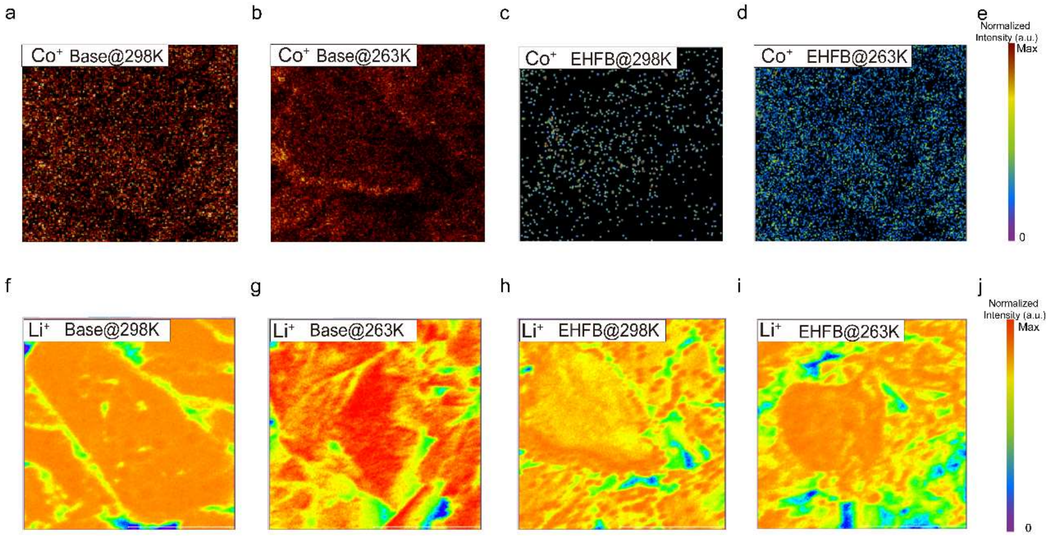

Supplementary Fig.30| The mapping of Co^+ (a-d) and Li^+ (f-i) distributed at the surface of the graphite anode (FOV: 20μm x 20μm, Scale Bar: 10μm). The scalebar of normalized intensity for Co^+ distribution (e) and for Li^+ distribution (j).

There detected a serious Co^+ dissolution on the graphite anode cycled in Base electrolyte due to the high voltage electrochemical window, which is supposed to be inhibited when cycled in EHFB electrolyte because introducing fluorine can enhance the HOMO energies and thus improve the oxidation stability. The Li^+ distribution of cycled in Base electrolyte at LT shows much more than that in EHFB, which also manifests that the lithium dendrite is likely to growth at the graphite anode with Base electrolyte during LT operations.

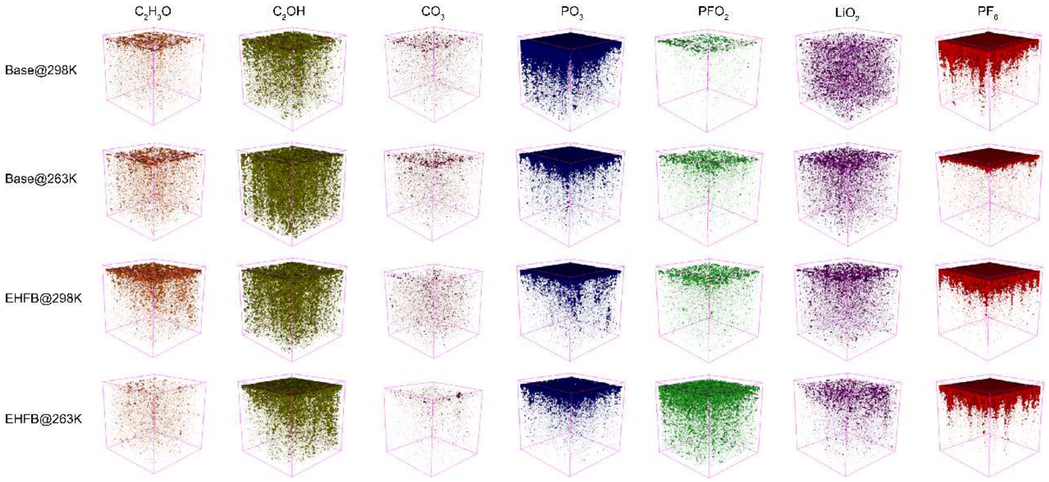

Supplementary Fig.31| The mapping of Co^{+} and Li^{+} distributed at the surface of the graphite anode.

For the organic component C-H-O, it shows more in the outer of the SEI layer, and it deceases in EHFB cells but increases in Base cells when the temperature jumps from RT to LT. For the inorganic component, it shows much more P-F-O and residue PF_6^- and less Li_2O in EHFB cells at LT. And it was found that the lithium salt was evenly distributed at the outer SEI layer.

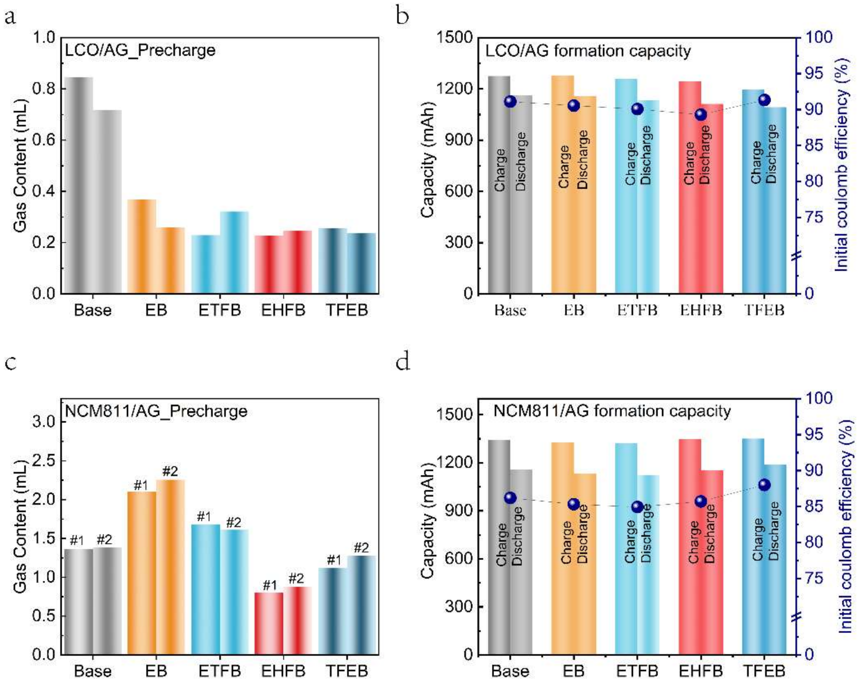

Supplementary Fig.32|Formation data of the pouch cells for NCM811/Gr (a-b) and LCO/Gr (c-d). The gas production amount (a,c) and the capacity during formation process (b,d).

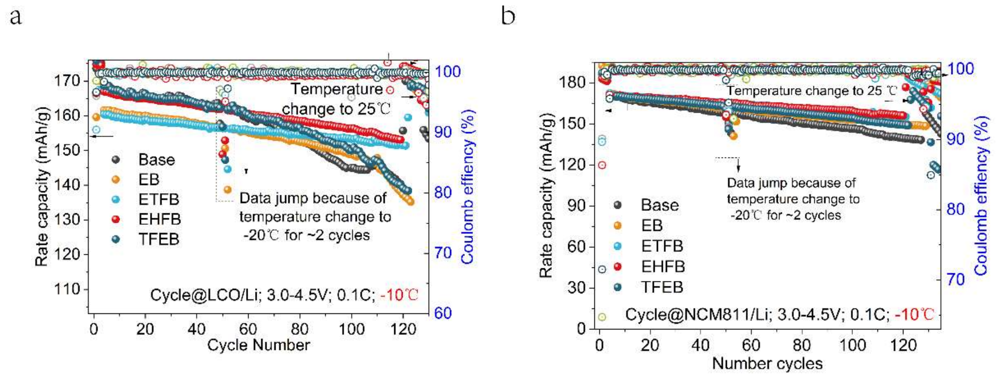

Supplementary Fig.33| Cycling behavior of half-cells with the electrochemical window of 3-4.5V. (a) and NCM811/Li half-cells (b) LCO/Li under −10 °C.

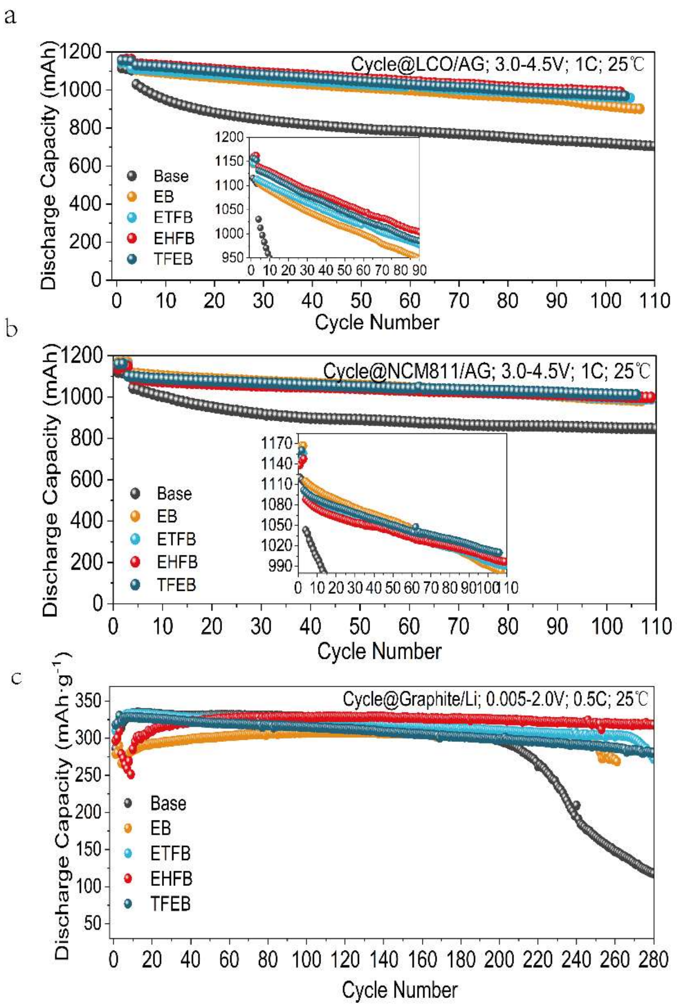

Supplementary Fig.34| Cycling behavior of pouch cells under room temperature. a, 4.5V LCO/Gr. b, 4.5V NCM811/Gr. c, Gr/Li half-cell.

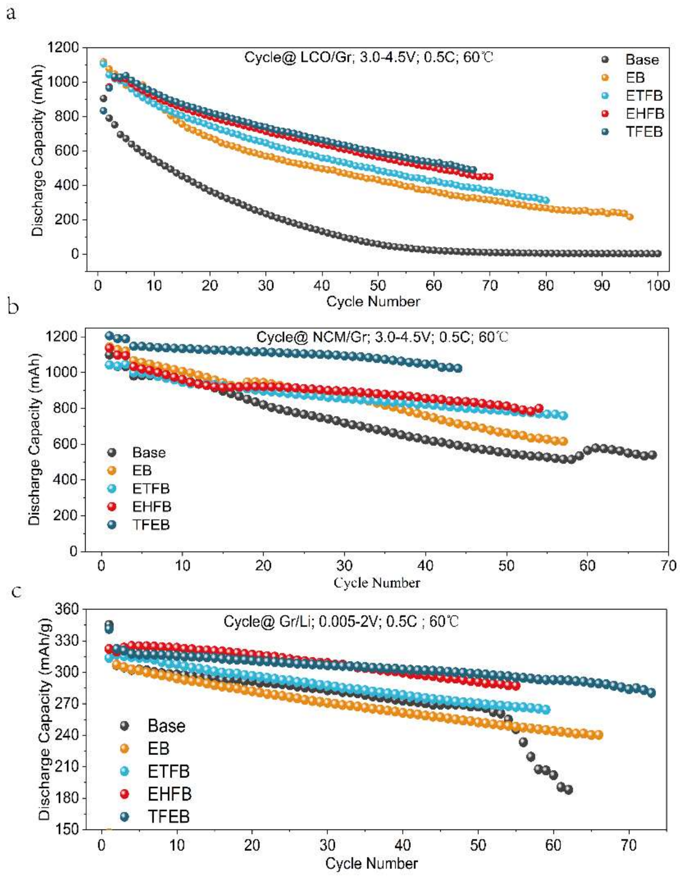

Supplementary Fig.35| Cycling behavior of pouch cells under high temperature of 60 °C. a, 4.5V LCO/Gr. b, 4.5V NCM811/Gr. c, Gr/Li half-cell.

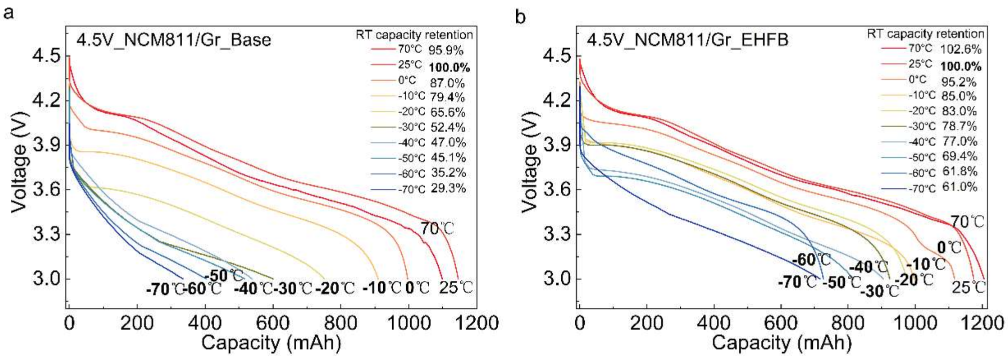

Supplementary Fig.36| Temperature dependent discharge profiles of NCM811/Gr pouch cells. a, Base. b, EHFB.

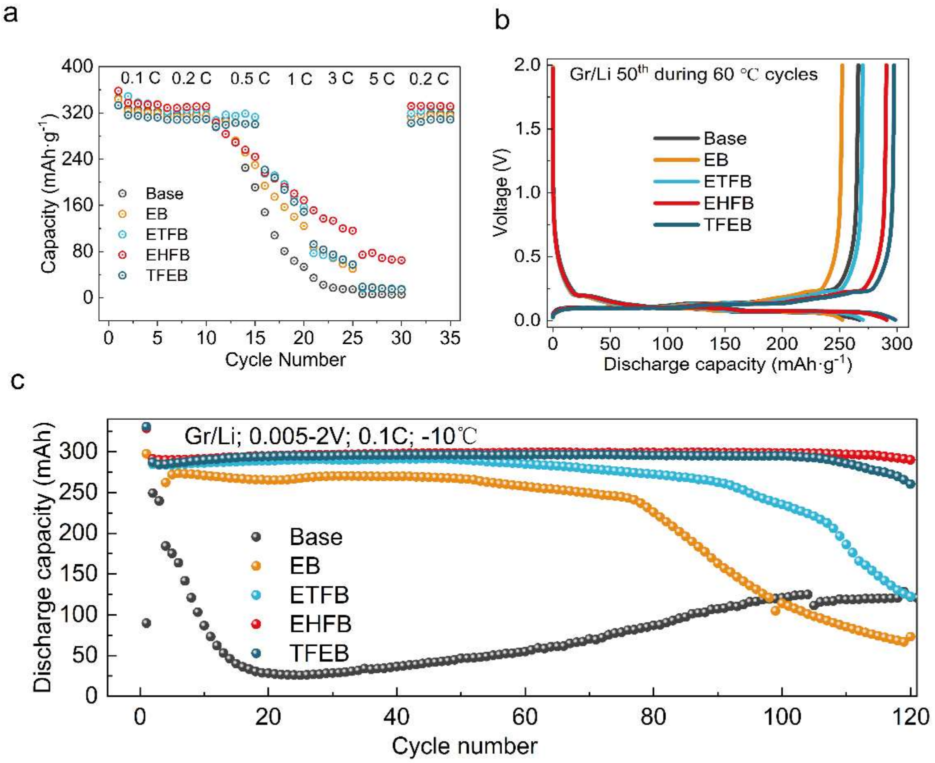

Supplementary Fig.37| Electrochemical behavior of graphite cells. a, Rate performance of Gr/Li cells. b, 50th charge and discharge profiles of Gr/Li cells during 60 °C cycles. c, Cycling performance of Gr/Li cell under −10 °C with different electrolytes.

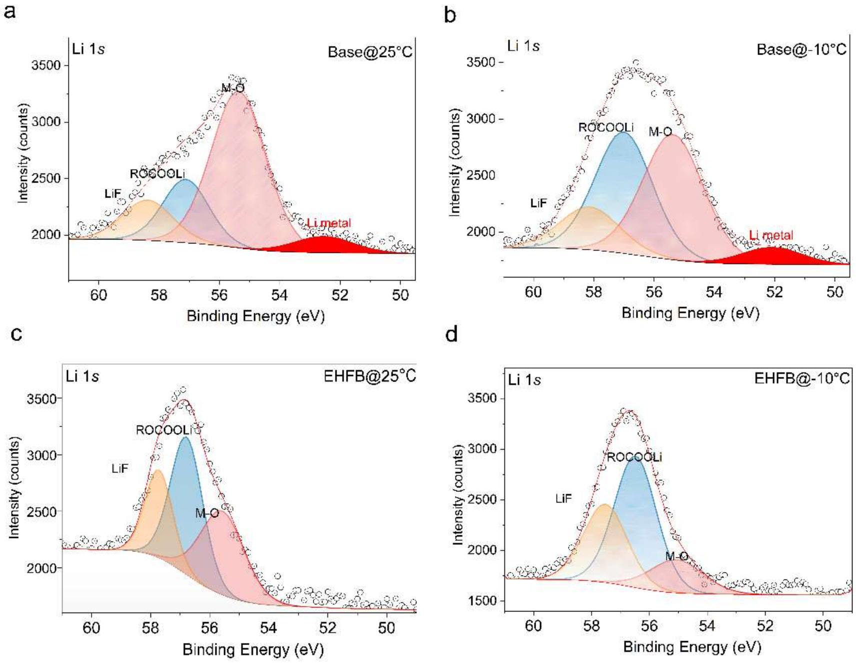

Supplementary Fig.38| X-ray photoelectron spectroscopy (XPS) profiles of Li 1*s* of the dismantled graphite anode after the long cycling for both electrolytes at −10 °C and 25 °C. (a-b) Base electrolyte and (c-d) EHFB electrolyte under 25°C (a-c) and −10°C (b-d).

Based on the XPS profile analysis, a distinct peak corresponding to metallic lithium (~52.2 eV)[37, 38] was observed on the surface of graphite electrodes cycled in the base electrolyte (Supplementary Fig. 38). In contrast, no such peak was detected on the surface of graphite electrodes cycled in the EHFB electrolyte, even at low temperatures. These XPS findings align with the results obtained from XRD analysis, providing compelling evidence that the EHFB electrolyte effectively addresses the dendrite formation issue during low-temperature cycling, which was supplementarily discussed in the manuscript.

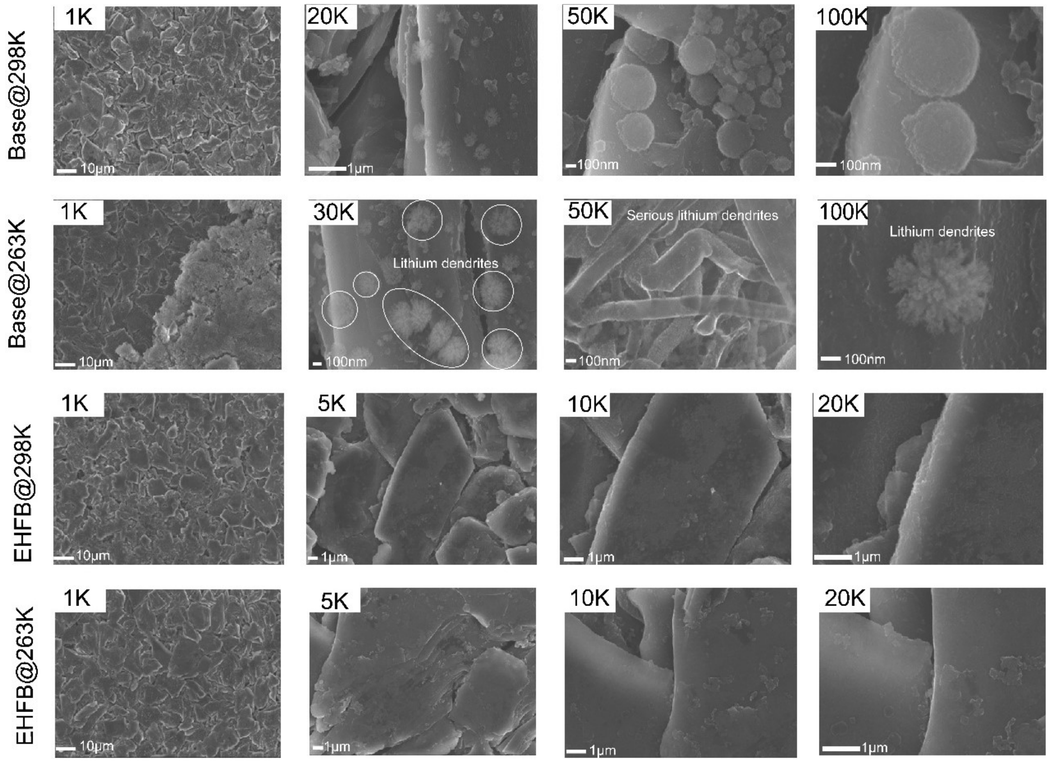

Supplementary Fig.39| SEM images of graphite anode dismantled from LCO/Gr pouch cell after long cycling at Base and EHFB electrolyte under different temperature.

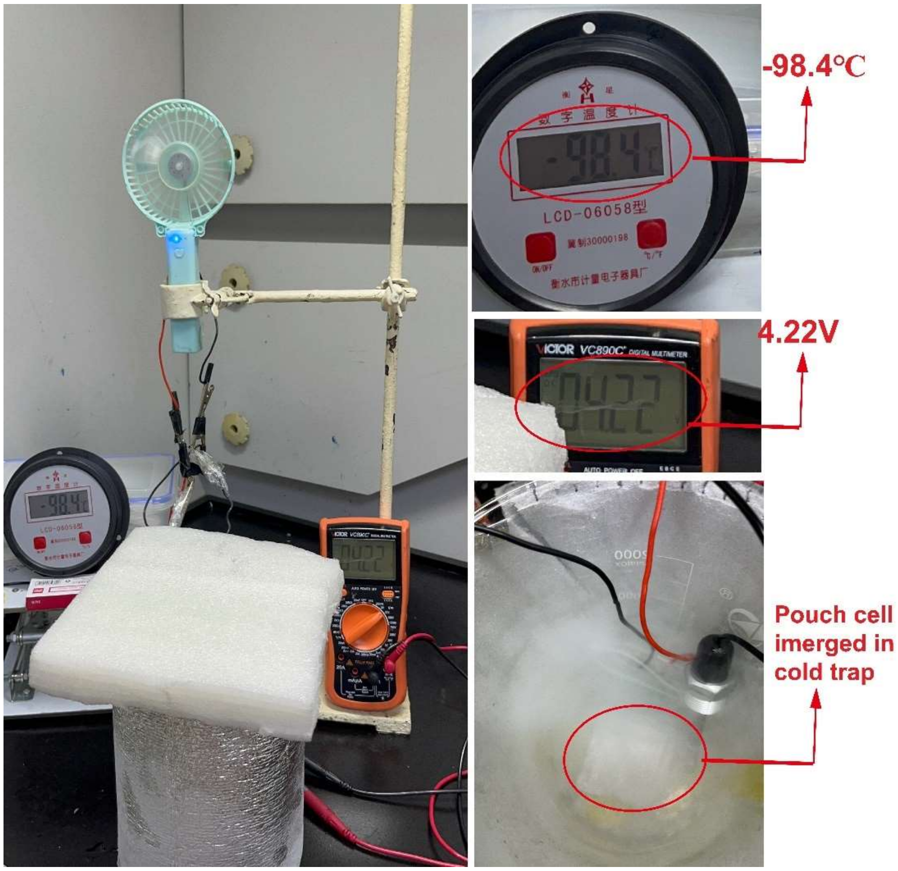

Supplementary Fig.40| Photos of an electric fan powered by the 4.5V LCO/Gr pouch cell using EHFB electrolyte at superior low temperature −98.4 °C

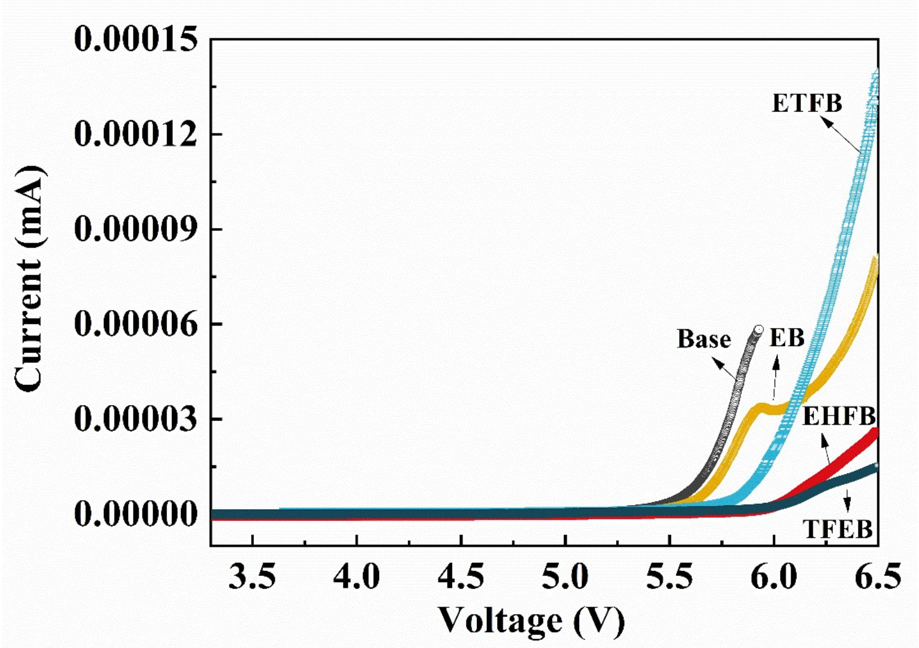

Supplementary Fig.41| LSV of the electrolyte with a scan rate of 0.2mV/s with base electrolyte, EB, ETFB, EHFB, and TFEB contained electrolyte.

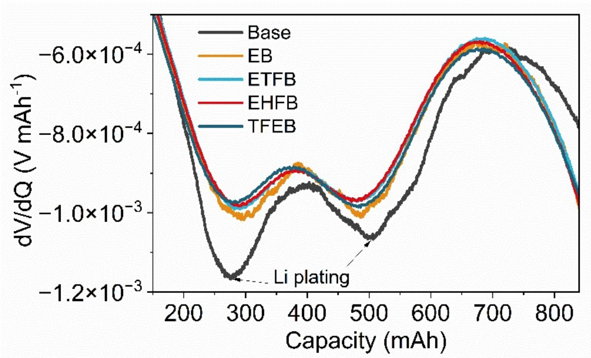

Supplementary Fig.42| Differential voltage (dV/dQ) profiles of the 100th discharge curve cycled at −10 °C.

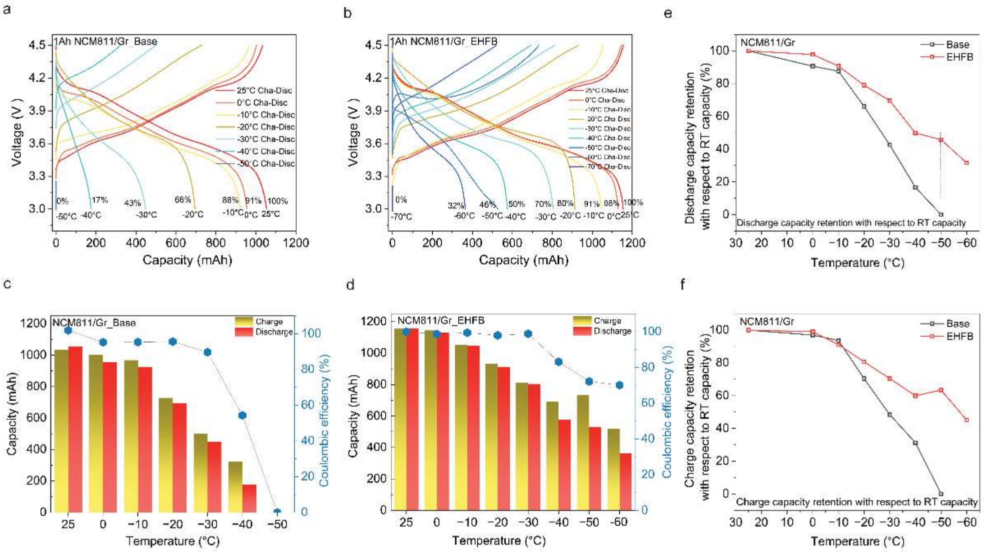

Supplementary Fig.43| a-b, temperature dependent charge and discharge profiles of 4.5V NCM811/Gr pouch cell using base (a) and EHFB electrolyte (b) under low temperature charge-discharge mode. c-d, the corresponding charge and discharge capacity and coulombic efficiency under different temperature. e-f, the capacity retention of room temperature at all temperatures for discharge (e) and charge (f).

*Note: The charge capacity showed slightly increased at -50 °C is due to a rest at room temperature for 1 hour before cycle at −50 °C, to relieve the alarm of long-term low temperature operation of the test chamber.

The NCM811/Gr cell utilizing EHFB electrolyte demonstrates significantly enhanced charge and discharge capacities at each temperature, compared to the base electrolyte. Specifically, when charged at −30°C, the NCM811/Gr cell with the base electrolyte achieves only 43% of its room temperature capacity (448mAh). In contrast, the cell using EHFB electrolyte attains a discharge capacity of 70% of its room temperature capacity (804mAh) under the same conditions. Furthermore, at −40°C, the cell using the base electrolyte nearly fails, exhibiting a discharge

capacity of merely 17% of its room temperature capacity. Conversely, the NCM811/Gr cell with EHFB electrolyte still maintains a discharge capacity of 46% of its room temperature capacity after charging at −50°C. Surprisingly, even at an extremely low temperature of −60°C, the cell using EHFB electrolyte continues to display remarkable performance, showcasing a charging capacity of 520mAh and a discharge capacity of 364.6mAh.

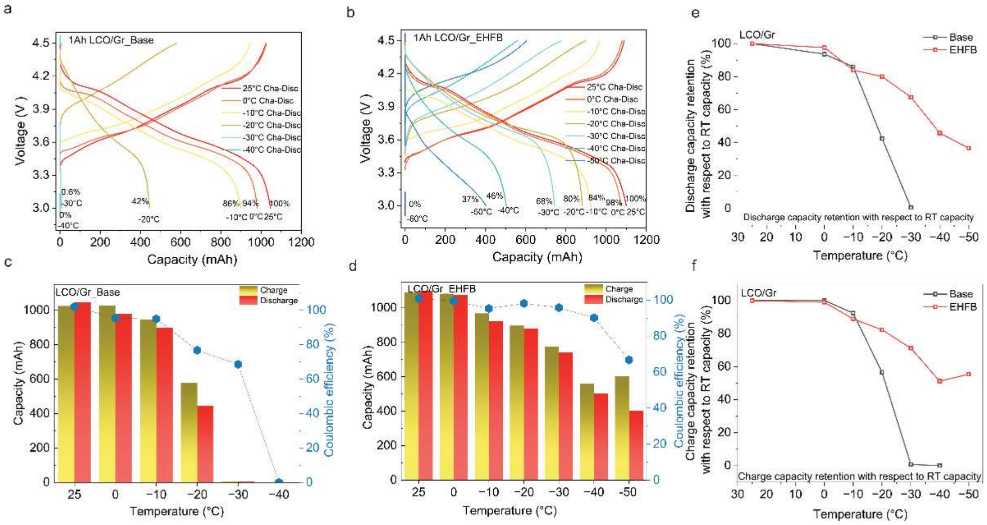

Supplementary Fig.44| a-b, temperature dependent charge and discharge profiles of 4.5V LCO/Gr pouch cell using base (a) and EHFB electrolyte (b) under low temperature charge-discharge mode. c-d, the corresponding charge and discharge capacity and coulombic efficiency under different temperature. e-f, the capacity retention of room temperature at all temperatures for discharge (e) and charge (f).

The LCO/Gr cell utilizing the base electrolyte exhibited a considerable capacity decay at −20 °C, with a discharge capacity amounting to only 42% of the room temperature capacity. Eventually, at an extreme low temperature of −30 °C, the cell was rendered incapable of delivering any capacity. On the contrary, the LCO/Gr cell employing the EHFB electrolyte demonstrated improved performance at various temperatures. At −30 °C, it was able to achieve a discharge capacity of up to 68% of the room temperature capacity. Remarkably, even at the demanding temperature of −50 °C, it exhibited a charging capacity of 603mAh and a discharge capacity of 403mAh (37% of RT retention), highlighting its enhanced capabilities.

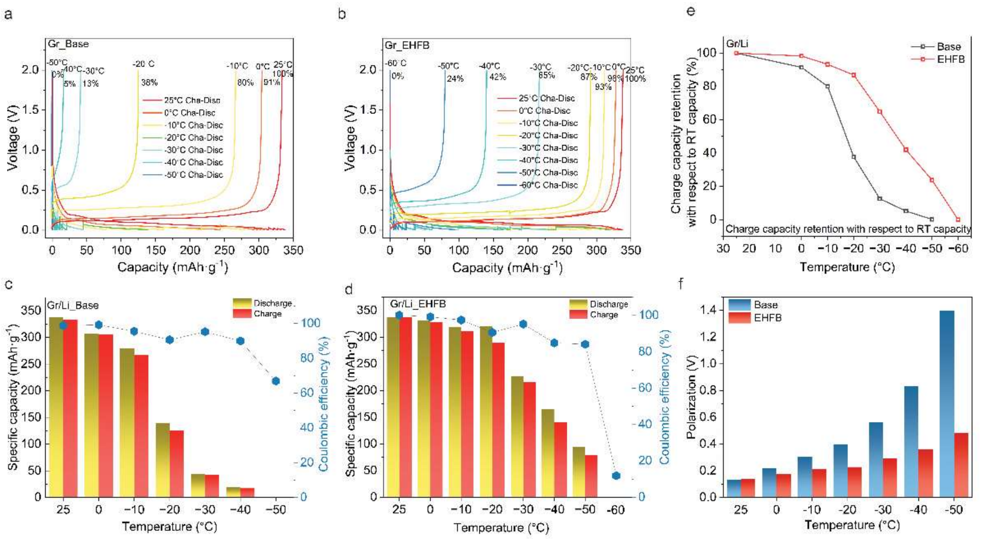

Supplementary Fig.45| a-b, temperature dependent charge and discharge profiles of Gr/Li half cells using base (a) and EHFB electrolyte (b) under low temperature charge-discharge mode. c-d, the corresponding charge and discharge capacity and coulombic efficiency under different temperature. e, the capacity retention of room temperature at all temperatures. f, the polarization voltage of Gr/Li cell using the two electrolytes.

The Gr/Li cells utilizing the base electrolyte experience a significant capacity decline at −20 °C, resulting in a discharge capacity of only 38% of the room temperature capacity. Moreover, these cells are unable to perform satisfactorily below −20 °C and the polarization increased sharply, indicating their limitations in low-temperature environments. In contrast, the Gr/Li cells cycled in the EHFB electrolyte exhibit excellent specific capacities across a range of temperatures. At −30 °C, the retention of room temperature capacity reaches an impressive 65%, highlighting the efficacy of the EHFB electrolyte in maintaining performance at sub-zero temperatures. Furthermore, even after charging at −40 °C, the cells can still discharge to 42% of the room temperature capacity, demonstrating their resilience under challenging conditions. Notably, at an even lower temperature of −50 °C, the Gr/Li cells using EHFB electrolyte deliver remarkable results, with a discharge specific capacity of 95mAh/g (24% of RT retention) and a charge specific capacity of 80mAh/g, with much smaller polarization increase. This showcases the superior performance achieved through the utilization of the EHFB electrolyte, particularly in extremely cold environments.

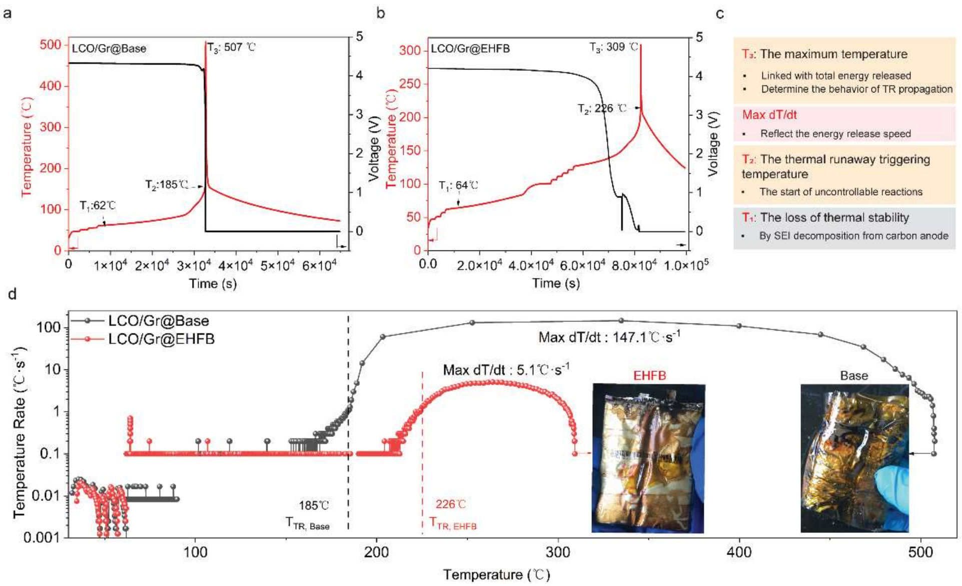

Supplementary Fig.46| Thermal safety evaluation of the 1Ah LCO/Gr pouch cell based on accelerating rate calorimetry (ARC). a-b) Temperature/ voltage profiles of the cell using Base electrolyte (a) and EHFB electrolyte (b). c) The explanation of the key point temperature during ARC test. d) Temperature independence of temperature rate of the fully charged cells. The insets show the photos after the test.

The pouch cell utilizing the Base electrolyte exhibited an alarming temperature rate of up to 147.1 °C s^{-1}. Surprisingly, when employing the EHFB electrolyte, the maximum temperature rate (max dT/dt) dropped to a much lower value of 5.1 °C s^{-1}. Notably, the temperature at which thermal runaway is triggered (T_{TR} = T_2) for the cell using EHFB electrolyte (226 °C) is substantially higher than that of the cell using Base electrolyte (185 °C), suggesting a greater tendency to avoid thermal runaway incidents. Additionally, the maximum temperature reached by the cell with Base electrolyte was 507 °C, whereas the cell with EHFB electrolyte exhibited a significantly lower maximum temperature (309 °C), indicating a considerable mitigation of the total energy released during the thermal runaway process. Taken together, these findings highlight the substantial improvement in safety provided by our EHFB electrolyte for the full cell configuration.

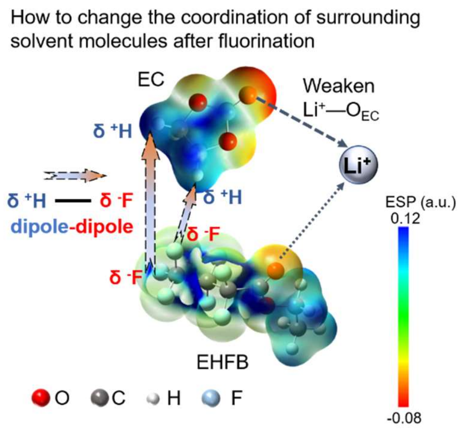

Supplementary Fig.47| The effect of fluorinated solvents on the dipole-dipole interactions among surrounding solvent molecules and their coordination with Li^+.

Why the fluorination affects the surrounding solvent can be explained as follows as illustrated in Fig S47: The introduction of fluorine atoms primarily induces a shift in the dipole moment within the molecule itself. However, the changes in charge distribution due to dipole modification also impact the electronegativity of the molecule and the surrounding molecules. The alteration in the coordination behavior of surrounding solvent molecules upon fluorination can be attributed to the dipole-dipole interactions between the solvent and surrounding solvent molecules. The electrostatic distribution of solvent molecules reveals an increased electronegativity at the fluorinated methylene end of the solvent, while the electronegativity of the surrounding solvent molecules remains biased towards the carbonyl oxygen end. Consequently, the dipole-dipole interactions between the non-fluorinated solvent molecules and the surrounding solvent molecules undergo changes after fluorination, as supported by findings in the literature[39]. These insights collectively indicate that fluorination significantly affects the coordination behavior of surrounding solvent molecules.

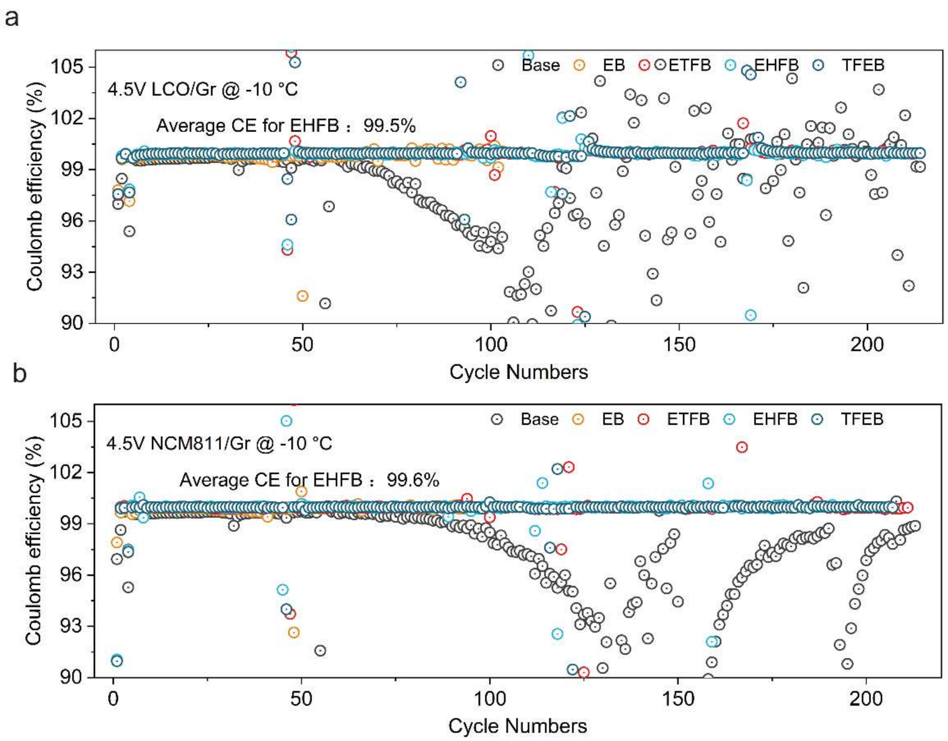

Supplementary Fig.48| Coulomb efficiency of the pouch cells cycling under −10 °C. a) LCO/Gr; b) NCM811/Gr

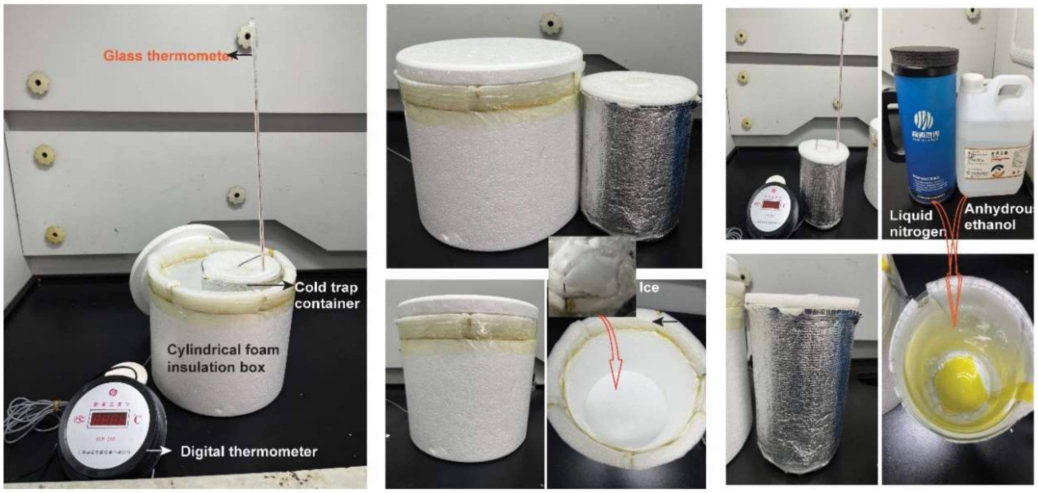

Supplementary Fig.49| The physical diagram of the advanced cold trap device for low temperature test.

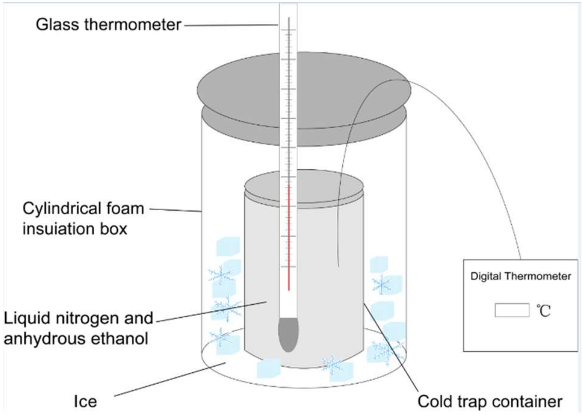

Supplementary Fig.50| Illustration of the cold trap device for low temperature test.

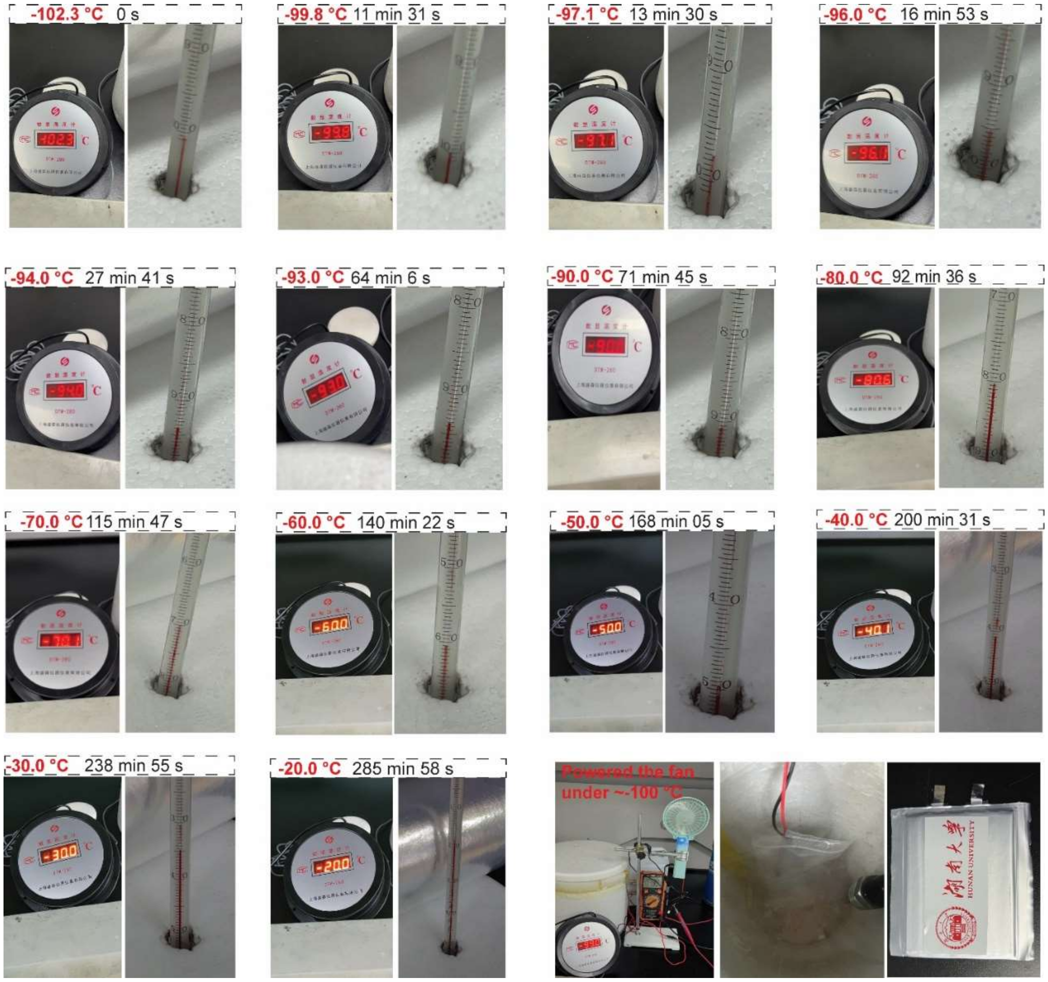

Supplementary Fig.51| Temperature monitoring in low-temperature test achieved through dual calibration with digital thermometer and glass thermometer to ensure the accuracy and stability of the temperature, the whole process was recorded in Supplementary Movie 3-11.

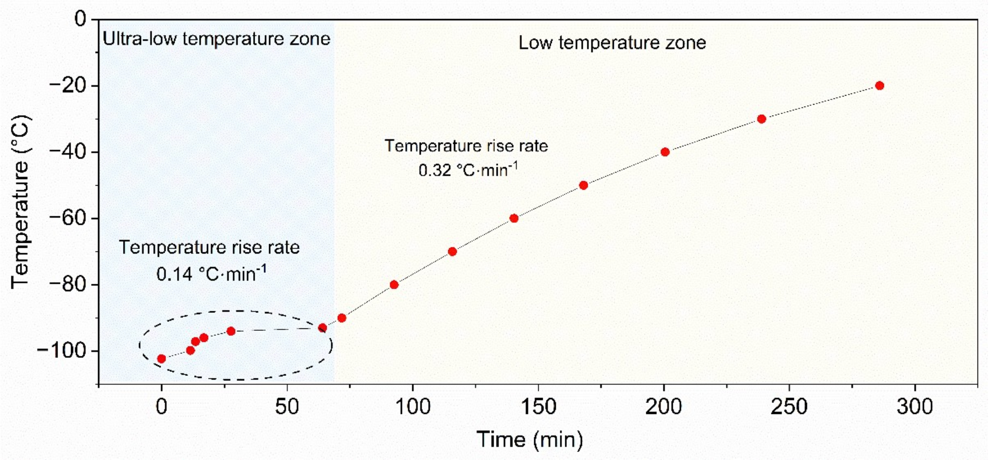

Supplementary Fig.52| The recording of temperatures changes with time of the cold trap (from ~−102 °C to −20 °C) during the low temperature test, in order to evaluate the accuracy and durability of the low temperatures, the whole process was recorded in Supplementary Movie 3-11.

The temperature rise rate in ultra-low temperature zone is only 0.14 $°C \cdot min^{-1}$, and that in low temperature zone is about 0.32 $°C \cdot min^{-1}$, which guarantees the accuracy and stability of the low temperature test, such as low temperature discharge and the low temperature conductivity.

Supplementary Table 10. The equilibrated dimensions and densities of the units of different solvation models.

	Base	EB	EHFB	ETFB	TFEB	Base	EHFB
			25 °C			−70 °C	
a (Å)	44.19959	43.87234	43.70915	44.02213	43.93867	43.320916	43.274098
Density (g/cm^3)	1.17	1.19	1.28	1.22	1.22	1.25	1.32

Reference

1. Fan X*, et al.* All-temperature batteries enabled by fluorinated electrolytes with non-polar solvents. *Nature Energy* 4, 882-890 (2019).

2. Akita Y, Segawa M, Munakata H, Kanamura K. In-situ Fourier transform infrared spectroscopic analysis on dynamic behavior of electrolyte solution on LiFePO4 cathode. *Journal of Power Sources* 239, 175-180 (2013).

3. Marino C*, et al.* Solvation and Dynamics of Lithium Ions in Carbonate-Based Electrolytes during Cycling Followed by Operando Infrared Spectroscopy: The Example of NiSb2, a Typical Negative Conversion-Type Electrode Material for Lithium Batteries. *The Journal of Physical Chemistry C* 121, 26598-26606 (2017).

4. Wu Y*, et al.* Significance of Antisolvents on Solvation Structures Enhancing Interfacial Chemistry in Localized High-Concentration Electrolytes. *ACS Central Science* 8, 1290-1298 (2022).

5. Lu W, Xie K, Chen Zx, Pan Y, Zheng Cm. Preparation and characterization of trifluoroethyl aliphatic carboxylates as co-solvents for the carbonate-based electrolyte of lithium-ion batteries. *Journal of Fluorine Chemistry* 161, 110-119 (2014).

6. Wang Z, Huang X, Chen L. Performance Improvement of Surface-Modified LiCoO2 Cathode Materials: An Infrared Absorption and X-Ray Photoelectron Spectroscopic Investigation. *Journal of The Electrochemical Society* 150, A199 (2003).

7. Yang CR, Wang YY, Wan CC. Composition analysis of the passive film on the carbon electrode of a lithium-ion battery with an EC-based electrolyte. *Journal of Power Sources* 72, 66-70 (1998).

8. Li J-T, Chen S-R, Ke F-S, Wei G-Z, Huang L, Sun S-G. In situ microscope FTIR spectroscopic studies of interfacial reactions of Sn–Co alloy film anode of lithium ion battery. *Journal of Electroanalytical Chemistry* 649, 171-176 (2010).

9. Ikezawa Y, Nishi H. In situ FTIR study of the Cu electrode/ethylene carbonate+dimethyl carbonate solution interface. *Electrochimica Acta* 53, 3663-3669 (2008).

10. Lu W, Xie K, Pan Y, Chen Z-x, Zheng C-m. Effects of carbon-chain length of trifluoroacetate co-solvents for lithium-ion battery electrolytes using at low temperature. *Journal of Fluorine Chemistry* 156, 136-143 (2013).

11. Aurbach D, Daroux ML, Faguy PW, Yeager E. Identification of Surface Films Formed on Lithium in Propylene Carbonate Solutions. *Journal of The Electrochemical Society* 134, 1611 (1987).

12. Li G, Li H, Mo Y, Chen L, Huang X. Further identification to the SEI film on Ag electrode in lithium batteries by surface enhanced Raman scattering (SERS). *Journal of Power Sources* 104, 190-194 (2002).

13. Perla B. Balbuena YW. Lithium-ion batteries: solid-electrolyte interphase.). Imperial College Press (2004).

14. Song W*, et al.* Electronic structure influences on the formation of the solid electrolyte interphase. *Energy & Environmental Science* 13, 4977-4989 (2020).

15. Wang Y, Nakamura S, Ue M, Balbuena PB. Theoretical Studies To Understand Surface Chemistry on Carbon Anodes for Lithium-Ion Batteries: Reduction Mechanisms of Ethylene Carbonate. *Journal of the American Chemical Society* 123, 11708-11718 (2001).

16. Xu M, Hao L, Liu Y, Li W, Xing L, Li B. Experimental and Theoretical Investigations of Dimethylacetamide (DMAc) as Electrolyte Stabilizing Additive for Lithium Ion Batteries. *The Journal of Physical Chemistry C* 115, 6085-6094 (2011).

17. Zhuang GV, Xu K, Yang H, Jow TR, Ross PN. Lithium Ethylene Dicarbonate Identified as the Primary Product of Chemical and Electrochemical Reduction of EC in 1.2 M LiPF6/EC:EMC Electrolyte. *The Journal of Physical Chemistry B* 109, 17567-17573 (2005).

18. Gauthier M*, et al.* Electrode–Electrolyte Interface in Li-Ion Batteries: Current Understanding and New Insights. *The Journal of Physical Chemistry Letters* 6, 4653-4672 (2015).

19. Lu W, Xie K, Chen Z, Xiong S, Pan Y, Zheng C. A new co-solvent for wide temperature lithium ion battery electrolytes: 2,2,2-Trifluoroethyl n-caproate. *Journal of Power Sources* 274, 676-684 (2015).

20. Campion CL, Li W, Lucht BL. Thermal Decomposition of LiPF6-Based Electrolytes for Lithium-Ion Batteries. *Journal of The Electrochemical Society* 152, A2327 (2005).

21. Kim K*, et al.* Cyclic Aminosilane-Based Additive Ensuring Stable Electrode–Electrolyte Interfaces in Li-Ion Batteries. *Advanced Energy Materials* 10, 2000012 (2020).

22. Plakhotnyk AV, Ernst L, Schmutzler R. Hydrolysis in the system LiPF6—propylene carbonate—dimethyl carbonate—H2O. *Journal of Fluorine Chemistry* 126, 27-31 (2005).

23. Yang H, Zhuang GV, Ross PN. Thermal stability of LiPF6 salt and Li-ion battery electrolytes containing LiPF6. *Journal of Power Sources* 161, 573-579 (2006).

24. Heider U, Oesten R, Jungnitz M. Challenge in manufacturing electrolyte solutions for

lithium and lithium ion batteries quality control and minimizing contamination level. *Journal of Power Sources* 81-82, 119-122 (1999).

25. Henschel J, Peschel C, Klein S, Horsthemke F, Winter M, Nowak S. Clarification of Decomposition Pathways in a State-of-the-Art Lithium Ion Battery Electrolyte through 13C-Labeling of Electrolyte Components. *Angewandte Chemie International Edition* 59, 6128-6137 (2020).

26. Murakami M, Yamashige H, Arai H, Uchimoto Y, Ogumi Z. Association of paramagnetic species with formation of LiF at the surface of LiCoO2. *Electrochimica Acta* 78, 49-54 (2012).

27. Wang A, Kadam S, Li H, Shi S, Qi Y. Review on modeling of the anode solid electrolyte interphase (SEI) for lithium-ion batteries. *npj Computational Materials* 4, 15 (2018).

28. Wilken S, Treskow M, Scheers J, Johansson P, Jacobsson P. Initial stages of thermal decomposition of LiPF6-based lithium ion battery electrolytes by detailed Raman and NMR spectroscopy. *RSC Advances* 3, 16359-16364 (2013).

29. Xu G, Liu Z, Zhang C, Cui G, Chen L. Strategies for improving the cyclability and thermo-stability of LiMn2O4-based batteries at elevated temperatures. *Journal of Materials Chemistry A* 3, 4092-4123 (2015).

30. Ye C*, et al.* Converting detrimental HF in electrolytes into a highly fluorinated interphase on cathodes. *Journal of Materials Chemistry A* 6, 17642-17652 (2018).

31. Illig J, Ender M, Weber A, Ivers-Tiffée E. Modeling graphite anodes with serial and transmission line models. *Journal of Power Sources* 282, 335-347 (2015).

32. Illig J, Schmidt JP, Weiss M, Weber A, Ivers-Tiffée E. Understanding the impedance spectrum of 18650 LiFePO4-cells. *Journal of Power Sources* 239, 670-679 (2013).

33. Schmidt JP, Berg P, Schönleber M, Weber A, Ivers-Tiffée E. The distribution of relaxation times as basis for generalized time-domain models for Li-ion batteries. *Journal of Power Sources* 221, 70-77 (2013).

34. Lu Y*, et al.* The carrier transition from Li atoms to Li vacancies in solid-state lithium alloy anodes. *Science Advances* 7, eabi5520 (2021).

35. Chen X, Li L, Liu M, Huang T, Yu A. Detection of lithium plating in lithium-ion batteries by distribution of relaxation times. *Journal of Power Sources* 496, 229867 (2021).

36. Zhou X, Huang J, Pan Z, Ouyang M. Impedance characterization of lithium-ion batteries aging under high-temperature cycling: Importance of electrolyte-phase diffusion.

Journal of Power Sources 426, 216-222 (2019).

37. Kanamura K, Shiraishi S, Tamura H, Takehara Zi. X-Ray Photoelectron Spectroscopic Analysis and Scanning Electron Microscopic Observation of the Lithium Surface Immersed in Nonaqueous Solvents. *Journal of The Electrochemical Society* 141, 2379 (1994).

38. Otto S-K, *et al.* In-Depth Characterization of Lithium-Metal Surfaces with XPS and ToF-SIMS: Toward Better Understanding of the Passivation Layer. *Chemistry of Materials* 33, 859-867 (2021).

39. Sun Q, *et al.* Dipole–Dipole Interaction Induced Electrolyte Interfacial Model To Stabilize Antimony Anode for High-Safety Lithium-Ion Batteries. *ACS Energy Letters* 7, 3545-3556 (2022).
