# Supplementary material for: Breaking solvation dominance of ethylene carbonate via molecular charge engineering enables lower temperature battery
Source: Nat Commun. 2023 Dec 14;14:8326. doi: 10.1038/s41467-023-43163-9 (PMC10721867; doi:10.1038/s41467-023-43163-9)
Supplement: Supplementary file 3 — Description of Additional Supplementary Files [file 41467_2023_43163_MOESM3_ESM.pdf]

Description of Additional Supplementary Files

Supplementary Movie 1: Electric fan powered by the 4.5V LCO/Gr pouch cell using EHFB electrolyte at almost -100 °C

Supplementary Movie 2: Electric fan failed to be powered by the 4.5V LCO/Gr pouch cell using Base electrolyte at -86 °C

Supplementary Movie 3: Record of electric fan powered by the 4.5V LCO/Gr pouch cell using EHFB electrolyte at temperature from -102 °C to -99.8 °C with advanced cold trap system

Supplementary Movie 4: Record of temperature variety from -100 °C to-90 °C with advanced cold trap system

Supplementary Movie 5: Record of temperature variety from -90 °C to-80 °C with advanced cold trap system

Supplementary Movie 6: Record of temperature variety from -80 °C to-70 °C with advanced cold trap system

Supplementary Movie 7: Record of temperature variety from -70 °C to-60 °C with advanced cold trap system

Supplementary Movie 8: Record of temperature variety from -60 °C to-50 °C with advanced cold trap system

Supplementary Movie 9: Record of temperature variety from -50 °C to-40 °C with advanced cold trap system

Supplementary Movie 10: Record of temperature variety from -40 °C to-30 °C with advanced cold trap system

Supplementary Movie 11: Record of temperature variety from -30 °C to-20 °C with advanced cold trap system
